# Glucagon-like peptide 1 (GLP-1) receptor agonists in experimental Alzheimer’s disease models: a systematic review and meta-analysis of preclinical studies

**DOI:** 10.3389/fphar.2023.1205207

**Published:** 2023-09-13

**Authors:** Fanjing Kong, Tianyu Wu, Jingyi Dai, Zhenwei Zhai, Jie Cai, Zhishan Zhu, Ying Xu, Tao Sun

**Affiliations:** ^1^ School of Intelligent Medicine, Chengdu University of Traditional Chinese Medicine, Chengdu, China; ^2^ School of Acupuncture-Moxibustion and Tuina, Chengdu University of Traditional Chinese Medicine, Chengdu, China; ^3^ Hospital of Chengdu University of Traditional Chinese Medicine, Chengdu, China; ^4^ State Key Laboratory of Southwestern Chinese Medicine Resources, School of Pharmacy, Chengdu University of Traditional Chinese Medicine, Chengdu, China

**Keywords:** glucagon-like peptide 1 receptor agonists, Alzheimer’s disease, animal models, neuroprotective effects, meta-analysis, systematic review

## Abstract

Alzheimer’s disease (AD) is a degenerative disease of the nervous system. Glucagon-like peptide-1 receptor agonists (GLP-1 RAs), a drug used to treat type 2 diabetes, have been shown to have neuroprotective effects. This systematic review and meta-analysis evaluated the effects and potential mechanisms of GLP-1 RAs in AD animal models. 26 studies were included by searching relevant studies from seven databases according to a predefined search strategy and inclusion criteria. Methodological quality was assessed using SYRCLE’s risk of bias tool, and statistical analysis was performed using ReviewManger 5.3. The results showed that, in terms of behavioral tests, GLP-1 RAs could improve the learning and memory abilities of AD rodents; in terms of pathology, GLP-1 RAs could reduce Aβ deposition and phosphorylated tau levels in the brains of AD rodents. The therapeutic potential of GLP-1 RAs in AD involves a range of mechanisms that work synergistically to enhance the alleviation of various pathological manifestations associated with the condition. A total of five clinical trials were retrieved from ClinicalTrials.gov. More large-scale and high-quality preclinical trials should be conducted to more accurately assess the therapeutic effects of GLP-1 RAs on AD.

## 1 Introduction

Alzheimer’s disease (AD), a chronic neurodegenerative disease, usually manifests clinically as significant amnestic cognitive impairment and, to a lesser extent, as non-amnestic cognitive impairment (Knopman et al., 2021). As the global population ages, the prevalence of AD has increased dramatically and has become the fourth leading cause of human death (Prince et al., 2015). According to surveys, the prevalence of dementia worldwide is estimated to rise to 131.5 million people by 2050 (Shah et al., 2016). While causing suffering for patients and families, it also imposes a severe economic burden on society. AD has become one of the most serious, expensive and burdensome diseases of this century (Scheltens et al., 2021).

AD is a complex multifactorial disease, and the specific pathogenesis is still unclear (Querfurth and LaFerla, 2010). The drugs currently approved for treating AD mainly target cholinergic and glutamatergic neurotransmission, such as donepezil, rivastigmine, galantamine, and memantine. Although these drugs may improve symptoms to some extent, they do not alleviate or stop disease progression (Mangialasche et al., 2010). Furthermore, almost all current attempts to design effective drugs have ultimately failed (Jeremic et al., 2021). It has become extremely urgent to find new drugs to treat AD effectively. The lack of improved treatments for the disease, as well as the difficulty and cost of developing new drugs have drawn attention to existing drugs for other indications (Ferrari et al., 2022). Type 2 diabetes mellitus (T2DM) and AD have overlapping pathophysiological mechanisms, including insulin resistance, inflammation, oxidative stress, altered glucose metabolism, etc., (Watson and Craft, 2003; Ferrari et al., 2022). As early as the 1980s, Siegfried Hoyer had already realized that brain glucose and energy metabolism were the sites of major abnormalities in AD (Hoyer et al., 1988). Cognitive impairment could be caused by insulin resistance in the AD brain, which is similar to patients with T2DM, and some scholars even believe AD may be considered type 3 diabetes (Steen et al., 2005; Stanley et al., 2016). Several studies have shown that antidiabetic drugs may exert neuroprotective effects by alleviating insulin resistance, reducing tissue inflammation, in addition to counteracting potentially harmful metabolic and vascular changes in the brain (Patrone et al., 2014). Hence, the application of drugs for T2DM in the treatment of AD seems to be a new and prospective approach.

Glucagon-like peptide 1 (GLP-1), an incretin hormone, can enhance glucose-dependent insulin secretion and lower blood glucose (Kieffer and Habener, 1999; Du et al., 2010), which is widely used to treat T2DM. According to research, GLP-1 receptors are broadly distributed in the central nervous system (CNS), particularly in the hypothalamus and hippocampus (Alvarez et al., 2005; Egefjord et al., 2012; Campbell and Drucker, 2013). When activated, the receptor can heighten neuroexcitability and improve cognitive performance (During et al., 2003; Abbas et al., 2009; Hamilto and Holscher, 2009). Unfortunately, with a rapid degradation by dipeptidyl peptidase IV (DPPIV), natural GLP-1 peptide has a half-life of only 2–3 min in plasma, affecting clinical applications (Deacon et al., 1995). To address this phenomenon, glucagon-like peptide 1 receptor agonists (GLP-1 RAs), whose function similarly to GLP-1 but have a longer half-life, have been developed. GLP-1 RAs was originally used to treat type 2 diabetes and currently approved drugs include exenatide, liraglutide, lixisenatide, dulaglutide, semaglutide, albiglutide (discontinued in July 2017), etc., (Trujillo et al., 2021). In the past few years, the GLP-1 RAs as a potential therapy has been evaluated in a several studies for ameliorating neurodegenerative disease (Bassil et al., 2014; Talbot, 2014). Notably, GLP-1 RAs have displayed neuroprotective effects in a variety of AD preclinical models, particularly in models with the deposition of β-amyloid (Aβ) (Querfurth and LaFerla, 2010). However, these studies are still in an initial stage, and have a long way to go to fully validate their therapeutic effects in AD.

This paper presents a meta-analysis and systematic review of studies on GLP-1 RAs treatment in AD animal models. We summarize the effects of GLP-1 RAs on behavioral tests and main pathological features (Aβ and phosphorylated tau), and discuss the therapeutic mechanisms. These results provide insight into the GLP-1 RAs ameliorating AD, and a reference for further research and clinical translation.

## 2 Methods

### 2.1 Review protocol

This study was based on the *Preferred Reporting Items for Systematic Reviews and Meta-Analyses* (PRISMA) and the Cochrane Collaboration. It has been registered in PROSPERO (CRD42022367674).

### 2.2 Search strategy

The MeSH terms “GLP-1 receptor agonists (GLP-1RAs)” and “Alzheimer’s Disease (AD)” were used as keywords to search in seven databases: PubMed, Web of Science, Embase, Cocharne library, China National Knowledge Infrastructure (CNKI), Wanfang Data Information Site, and China Science and Technology Journal Database (VIP). We searched for articles up to 20 September 2022. Two authors (Fanjing Kong and Tianyu Wu) independently judged the retrieved literature by reading the title, abstract and full text according to inclusion and exclusion criteria. Inconsistent results were discussed or decided by a third author (Tao Sun).

### 2.3 Inclusion and exclusion criteria

The inclusion criteria for AD animal experiments with GLP-1RAs were as follows:

(1) Object: rodents;
(2) Intervention type: the study must include GLP-1 RAs group and control group;
(3) Outcome indicators: behavioral test must include Morris water maze (MWM);
(4) Article type: articles without full text or original data were excluded.

### 2.4 Data extraction and quality assessment

The extracted information from the included studies was as follow: 1) basic information (first author, year of publication); 2) test animals (species, sex, age); 3) interventions (drug, dose, administration period); 4) experimental results (behavioral experiments, neuropathological change). When a study contained multiple outcomes, they were considered as independent data. Plot digitizer 1.3 software was applicated to extract data from the graphs. The risk of bias was assessed by the SYRCLE Risk of Bias (RoB) tool, primarily designed for animal research.

### 2.5 Statistical analysis

ReviewManager 5.3 software was applied to conduct meta-analysis of the study data. Data were continuous variables, described as mean and standard deviation (SD), and analyzed by calculating standard mean differences (SMD). The heterogeneity of study results was determined by Q-test and *I* ^*2*^ statistic: *I* ^*2*^ ≤ 40% means low heterogeneity; 30% < *I* ^*2*^ ≤ 60% means mild heterogeneity; *I* ^*2*^ ≥ 50% means a high degree of heterogeneity (Higgins and Green, 2011). A random effect model was chosen for analysis when the study with high heterogeneity. *p*-value <0.05 was considered statistically significant.

## 3 Results

### 3.1 Study selection

**FIGURE 1:**
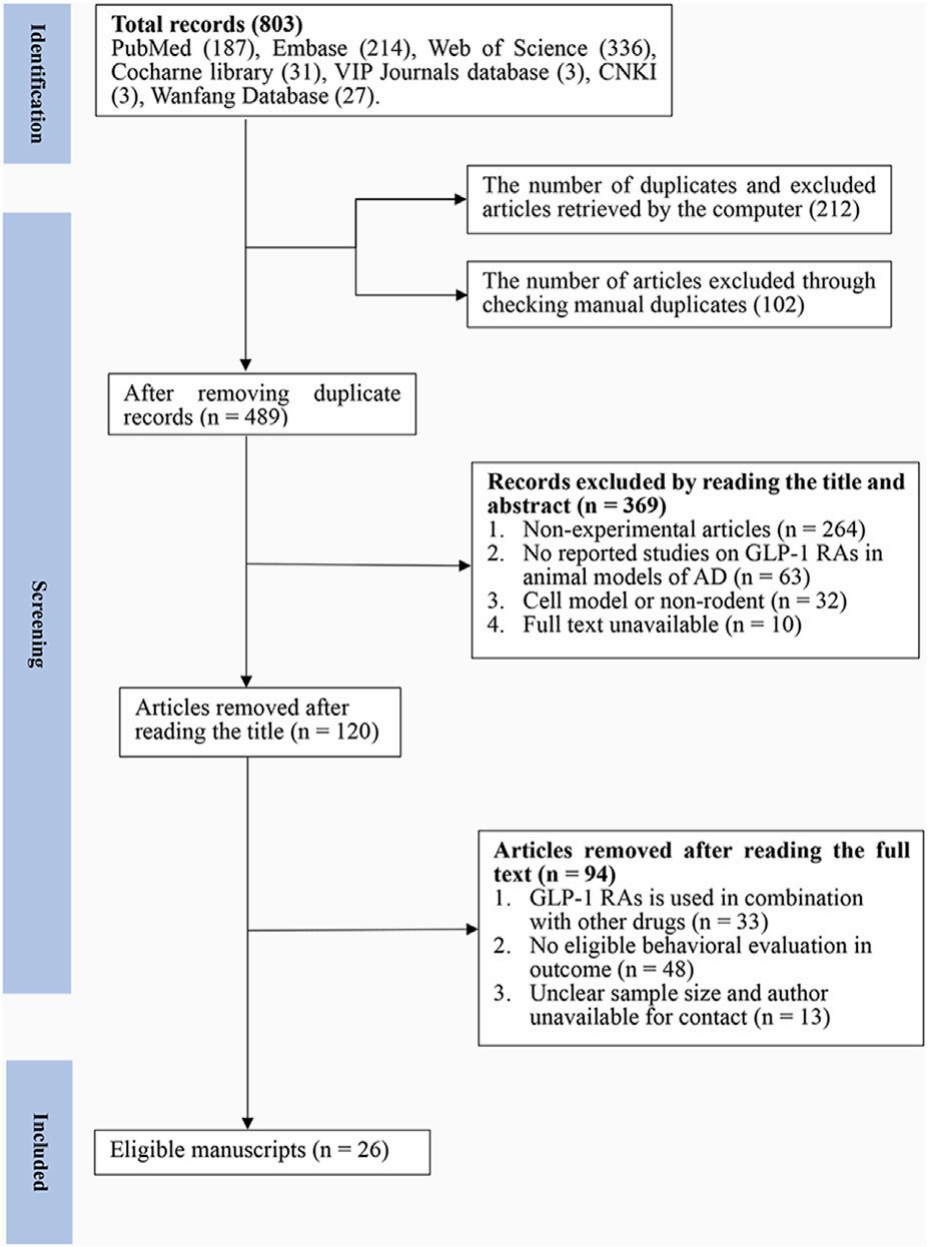
Flow diagram of the study selection process. 26 eligible articles were selected from 803 documents for a comprehensive analysis according to the predefined inclusion and exclusion criteria.

A total of 803 articles were retrieved from the seven databases, and 489 articles were identified after removing duplicates (*n* = 314). After browsing through the titles and abstracts, 369 papers were removed for at least one of the following reasons: 1) non-experimental articles (*n* = 264) 2) no reported studies of GLP-1 RAs on AD animal models (*n* = 63) 3) cell model or non-rodent (*n* = 32) 4) full text was not available (*n* = 10). Finally, after reading the full text, we selected 26 articles for analysis (Figure 1).

#### 3.1.1 Assessment of the quality of literature

**FIGURE 2:**
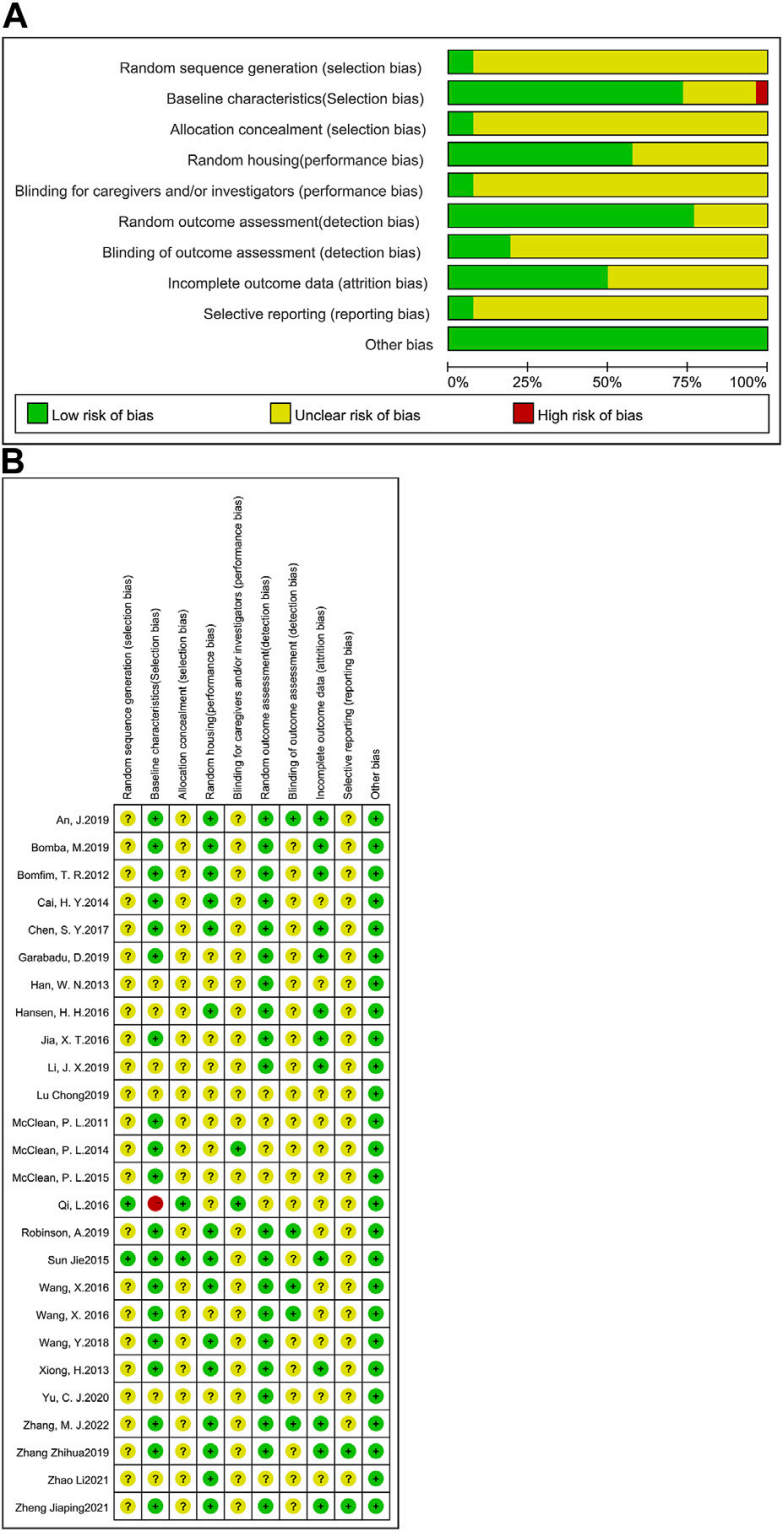
Assessment of literature quality using the SYRCLE risk of bias tool based on the Cochrane tool. **(A)** Risk of bias graph, **(B)** Risk of bias summary.

The RoB tool, developed by Hooijmans and other scholars (Hooijmans et al., 2014) from the SYRCLE Centre for Laboratory Animals in the Netherlands in 2014, was based on the Cochrane Randomized Controlled Trial Risk of Bias Assessment Tool. In this study, the RoB tool was selected to assess the risk of bias in the included studies. Here, a total of 26 studies were examined (Figure 2). Included articles were assessed independently by three authors (Tianyu Wu, Jie Cai and Zhishan Zhu), and where disagreements arose, they would be resolved after careful discussion with a fourth author (Ying Xu). The RoB tool consists of 10 entries, including selection bias, performance bias, detection bias, attrition bias, reporting bias and other biases. A report is considered low risk for its accordance with the entry requirements, high risk for no accordance, or ‘unclear’ for the reason that the reported details are insufficient. In this analysis, two studies described the detailed method of generating the allocation sequence and were seen as low risk of bias; the other 24 studies did not clarify the specific method of random allocation and were therefore classified as ‘unclear’. For baseline characteristics, more than half of the studies had similar baseline at the start of the experiment, so they were classified as ‘low risk’; seven studies did not specify and were classified as ‘unclear’. Except two studies, which described in detail the method of concealing allocation, the remaining studies were not clearly stated. 15 studies which clearly indicated randomized housing was judged to have a low risk of bias, whereas 11 studies that failed to determine randomized housing were categorized as having unclear risk of bias. Two studies clearly demonstrated that the results of the allocation were hidden for the investigators; thus, they were considered to be at low risk of bias; the other 24 studies were considered to be an unclear risk of bias. Five studies explicitly demonstrated the use of blinding of testers and were therefore considered to be at low risk of bias. 13 studies reported complete experimental results and were categorized as low risk. Studies that did not explicitly report allocation concealment, randomized outcome assessment and selective reporting were considered to be at an unspecified risk of bias. The results of the quality assessment suggest that the experimental design of animal studies should be detailed in the article in order to reduce the occurrence of unspecified risks.

#### 3.1.2 Study characteristics

In Table 1, we list the characteristics of these studies. Of the selected 26 articles, two kinds of animals were included: rats (*n* = 9) and mice (*n* = 17). And the strains of them included transgenic mice (*n* = 14), SD rats (*n* = 6), Wistar rats (*n* = 2), C57BL/6 mice (*n* = 2), Kunming mice (*n* = 1), C57BL/6 rats and (*n* = 1). There were 22 studies selecting male animals, two for both males and females, one for female animals, and one not mentioning the sex of the animals.

**TABLE 1:** Characteristics of the included studies.

Study (year)	Animal	Experimental group (drug, dose, duration, administration route)	Outcome		Mechanisms
			Behavioral change	Pathological change	
An, J. (2019)	5×FAD transgenic mice AD model (male, 5 months)	Exenatide	MWM	Aβ_1-42_ area↑; PSD95 and SYN↑; the numerical density of mitochondria; volume density of mitochondria; specific surface area of mitochondria↑; MDA↓ and SOD↑; ATP level↑; respiratory chain complex I activity↑; Drp1↓; Mfn2 and Opa1↑	Reduce Aβ_1-42_ deposition, alleviate synaptic degradation, improve mitochondrial morphology, relieve oxidative damage, correct the crisis of mitochondrial energy production, and normalize mitochondrial dynamics.
An et al. (2019)		100 μg/kg; twice a day for 16 weeks; s.c.			
Bomba, M. (2019)	3×Tg transgenic mice AD model (male and female, 6 months)	Exenatide	MWM, ORT	Aβ plaque; p-Tau; BDNF↑; pCREB↑; pTrkB↑; pERK5↑; pSyn↑; PSD95↑; pIRS-1↑; proBDNF↓; p75NTR↓; NF-κB; PPARs	Revert the adverse changes of BDNF signaling and the neuroinflammation status
Bomba et al. (2019)		500 μg/kg; 5 days per week for 3 months; i.p.			
Bomfim, T. R. (2012)	APP/PS1 transgenic mice AD model (male, 9 months)	Exenatide	MWM	IRS-1pSer636↓; IRS-1pSer312↓; p-JNK↓; plaque burden↓; soluble Aβ↓	Prevent disruption of brain insulin signaling in AD
Bomfim et al. (2012a)		25 nmol/kg; twice a day for 3 weeks; i.p.			
Cai, H. Y. (2014)	Aβ_25-35_-injected rat AD model (SD rats, male, adult, 200–230 g)	Lixisenatide	MWM	LTP↑; pGSK3β(S9) ↑; pGSK3β(Y216) ↓	Prevent Aβ-related impairments in synaptic plasticity and spatial memory (by affecting the PI3K-Akt-GSK3β pathway)
Cai et al. (2014)		10 nmol; once; i.h.			
Chen, S. Y. (2017)	3×Tg transgenic mice AD model (male, 7 months)	Liraglutide	MWM	tau↓; p-Tau (pt231, 217, ps396, 214, 199/20) ↓; phosphorylation or total NF-H and NF-M↓; p-ERK1/2↑; p-JNK/SAPKs↓	Reduce hyperphosphorylation of tau and NFs and reduce neuronal degeneration (by alterations in JNK and ERK signaling)
Chen et al. (2017)		300 μg/kg; once a day for 8 weeks; s.c.			
Garabadu, D. (2019)	Aβ-injected rat AD model (Wistar rats, male, 6–8 weeks, 160–200 g)	Exenatide	MWM, Y maze	level of Aβ_1-42_↓; ACh level↑; ChAT level↑; AChE level↑; the level of formazan produced in MTT assay↑; integrity in terms of the fluorescence intensity of TMRM↑; oxygen consumption↑; mitochondrial respiratory control ratio and ADP/O↑; mitochondrial complex enzyme-I, II, IV and V ↓; Akt and p-Akt↑	Mitigate AD-like manifestations including mitochondrial toxicity perhaps (by PI3K/Akt-mediated pathway)
Garabadu and Verma (2019)		5 μg/kg; once a day for 14 days; i.p.			
Han, W. N. (2013)	Aβ_25-35_-injected rat AD model (SD rats, male, adult, 230–250 g)	Liraglutide	MWM	L-LTP↑; cAMP↑	Improve spatial memory deficits and L-LTP deficits; activate the cAMP signaling pathway in the brain
Han et al. (2013)		0.05 nmol, 0.5 nmol, 5 nmol; once; i.h.			
Hansen, H. H. (2016)	hAPP Lon/PS1 A246E transgenic mice AD model (male, 5 months)	Liraglutide	MWM	Total brain volume; total plaque volume; total relative plaque load	N/A
Hansen et al. (2016)		100 μg/kg, 500 μg/kg; once a day for 3 months; s.c.			
Jia, X. T. (2016)	Aβ_1-42_-injected rat AD model (SD rats, male, adult, 230–250 g)	Exenatide	MWM	Bcl2↑; Caspase 3↓; Bax↓	Modulate the expression of Bcl2, Bax and caspase-3
Jia et al. (2016)		0.02 nmol, 0.2 nmol, 2 nmol; once; i.c.v.			
Li, J. X. (2019)	STZ-induced rat AD model (SD rats, male and female, 20–24 months, 320–400 g)	(Val8) GLP-1	MWM	MDA↓; SOD↑; AKT↑; ERK↑	Activate AKT/ERK signal transduction pathway (by strengthening the antioxidant activity of the brain tissue and reducing oxidative stress injury)
Li et al. (2019b)		5 μl; twice; i.c.v.			
McClean, P. L. (2011)	APPswe/PS1DE9 transgenic mice AD model (male, 7 months)	Liraglutide	MWM, ORT	LTP↑; PPF (paired-pulse facilitation) ↑; Aβ plaque↓; dense-core Congo red plaques↓; activated glia (Iba1 stain) ↓; young neurons (doublecortin-positive cells) ↑; Synaptophysin levels↑; Soluble Aβ oligomer and total APP levels↓	Prevent key neurodegenerative developments (prevented synapse loss and deterioration of synaptic plasticity in the hippocampus; enhance synaptic plasticity; reduce Aβplaque count and dense-core plaque numbers; reduce levels of soluble amyloid oligomers; reduce inflammation response; increase numbers of young neurons in the dentate gyrus)
McClean et al. (2011)		25 nm/kg; once a day for 8 weeks; i.p.			
McClean, P. L. (2014)	APPswe/PS1DE9 transgenic mice AD model (male, 14 months)	Liraglutide	MWM, ORT	LTP↑; Aβ plaque load↓; dense core plaque load↓; activated glia (Iba1 stain) ↓; new neurons (doublecortin-positive cells); soluble amyloid oligomer levels↓; total APP levels↓; synaptophysin levels↑; IDE levels↑; GSK3β levels↑; Nrk2b levels↑	Reverse some of the key pathological hallmarks of AD (reduce plaque load; reduce inflammation; increase neuronal progenitor cell count in the dentate gyrus; enhance LTP; increase synapse numbers; reduce total brain APP and beta-amyloid oligomer levels; increase IDE)
McClean and Hölscher (2014)		25 nm/kg; once a day for 8 weeks; i.p.			
McClean, P. L. (2015)	APPswe/PS1DE9 transgenic mice AD model (male, 8 weeks)	Liraglutide	MWM, ORT, open field	LTP↑; plaque load↓; dense-core Congo-red plaques↓; activated microglia (IBA-1 stain) ↓; young neurons (doublecortin-positive cells) ↑; Synaptophysin levels↑	Reduce the main hallmarks of AD (prevent synapse and synaptic plasticity loss; reduce amyloid plaque load and dense core congophilic plaques; reduce chronic inflammation; enhance neurogenesis)
McClean et al. (2015)		25 nm/kg; once a day for 8 months; i.p.			
Qi, L. (2016)	Aβ_1-42_-injected mice AD model (C57BL/6 mice, male, 8 weeks)	Liraglutide	MWM, Y maze	p-Tau↓; p-Akt↑; p-GSK3β↑; synaptic structure in the CA1 region↑; cell structure degradation↓	Decrease the phosphorylation of tau (via the protein kinase B and glycogen synthase kinase-3β pathways); alleviate the ultra-structural changes of pyramidal neurons and chemical synapses in the hippocampal CA1 region
Qi et al. (2016)		25 nmol/kg; once a day for 8 weeks; s.c.			
Robinson, A. (2019)	Tg2576 transgenic mice AD model (male, 4 months)	Exenatide	MWM	GSK3β↓; Aβ_1-42_; AKT1; IRS1; CTNNB1; INSR; AKT3; IGF1R	Reduced expression of IRSP genes, restore insulin signal transmission in brain
Robinson et al. (2019)		0.075μg; 6 days a week for 8 months; i.n.			
Wang, X. (2016)	Aβ_1-42_-injected rat AD model (SD rats, male, adult, 220–260 g)	Exenatide	MWM	LTP↑; cAMP↑; p-CREB↑	Modify impaired LTP; modifying the cAMP/PKA/CREB signaling pathway
Wang et al. (2016a)		0.2 nmol; once; i.h.			
Wang, X. (2016)	Aβ_31-35_-injected mice AD model (C57BL/6 mice, male, 8–10 weeks, 18–22 g)	Exenatide	Circadian rhythm, MWM, locomotor activity	N/A	N/A
Wang et al. (2016b)		0.05 nmol, 0.5 nmol; once; i.n.			
Wang, Y. (2018)	APP/PS1 transgenic mice AD model (male, 6 months, 30–45 g)	Exenatide	MWM	GnT-III ↓; aberrant bisecting GlcNAc levels↓; β-catenin↑; p-Akt↑; p-GSK3β↑; Aβ plaques↓; p-Tau↓	Downregulate aberrant GnT-III expression through the Akt/GSK-3β/β-catenin signaling pathway in neurons
Wang et al. (2018)		25 nmol/kg; once a day for 4 weeks; s.c.			
Xiong, H. (2013)	STZ-induced mice AD model (Kunming mice, male, 3 months, 38.66 ± 1.80 g)	Liraglutide	MWM	hyperphosphorylation of NFs↓; glycosylation level↑; p-Tau↓; microtubule binding tau↑; p-ERK1↑; p-JNK1↓; p-JNK2↓; degenerated neurons↓	Decrease p-Tau and neurofilament proteins by enhancing O-glycosylation of neuronal cytoskeleton protein, improving the JNK and ERK signaling pathway, and reducing neural degeneration
Xiong et al. (2013)		300 μg/kg; once a day for 30 days; s.c.			
Yu, C. J. (2020)	OA-induced rat AD model (C57BL/6 rats, female)	Liraglutide	MWM, Y maze	the level of neuronal apoptosis↓; p-Tau↓; BACE1↓	Prevent neuronal apoptosis and inhibit the activation of tau and the expression of BACE1
Yu et al. (2020)		300 μg/kg; once a day for 30 days; s.c.			
Zhang, M. J. (2022)	5×FAD transgenic mice AD model (male, 5 months)	Exenatide	MWM	Aβ_1-42_↓; NLRP2↓; GFAP↓; IL-1β↓; TNF-α↓; the number of NeuN-positive cells↑	Mitigate AD-related cognitive impairment and neuroinflammation by suppressing NLRP2 activation
Zhang et al. (2022)		100 μg/kg; twice a day for 16 weeks; s.c.			
Lu Chong (2019)	STZ-induced rat AD model (SD rats, male, 8–9 months, 200–240 g)	Liraglutide	MWM	Aβ_1-42_↓; PPARγ↑	Inhibit the accumulation of Aβ by activating PPARγ
Lu et al. (2019)		200 μg/kg; once a day for 28 days; i.p.			
Sun Jie (2015)	APP/PS1/Tau transgenic mice AD model (1 month, 10–14 g)	Liraglutide	MWM	Aβ_1-42_↓	Improve insulin signaling pathway and reduce Aβ
Sun et al. (2015)		300 μg/kg; once a day for 2 months; i.p.			
Zhang Zhihua (2019)	APP/PS1 transgenic mice AD model (male, 6 months, 25 ± 2.8 g)	Geniposide	Novel object recognition, MWM	number of neurons↑; senile plagues↓; Aβ_1-40_↓; Aβ_1-42_↓	Downregulation of mTOR signal pathway and enhancement of autophagy
Zhang (2019)		50 mg/kg; once a day for 6 weeks; i.g.			
Zhao Li (2021)	STZ-induced rat AD model (wistar rats, male, 180–220 g)	Liraglutide	MWM	p-Tau↓	Decrease the expression of p-Tau in hippocampus
Zhao et al. (2021)		300 μg/kg; once a day for 4 weeks; s.c.			
Zheng Jiaping (2021)	5×FAD transgenic mice AD model (male, 4 months)	Liraglutide	MWM	Aβ accumulation↓; the number of neurons↑; morphology of synapses; PSD95↑and SYN↑; GFAP↓; glycolytic enzymes (HIF-1α, HK1, PKM1/2, PKM2, PFKFB3) ↑; PDK2↑; p-PDH↓; ROS↓; GSH↑; ATP↑; p-PI3K↑; p-AKT↑	Reduce oxidative stress and improve astrocyte’s neuronal supportive functions
Zheng (2021)		25 μg/kg; once a day for 8 weeks; s.c.			

Note: Aβ, β-amyloid; MWM, morris water maze; PSD95, postsynaptic density protein 95; SYN, synaptophysin; MDA, malondialdehyde; SOD, superoxide dismutase; ATP, adenosine triphosphate; Drp1, dynamin-related protein 1; Mfn2, mitofusin 2; Opa1, optic atrophy protein 1; BDNF, brain-derived neurotrophic factor; pCREB, phosphorylated cAMP response element-binding protein; pTrkB, phosphorylated tropomyosin receptor kinase B; pERK5, phosphorylated extracellular signal-regulated kinase 5; IRS, insulin receptor substrate; NF-κB, nuclear factor kappa B; PPARs, peroxisome proliferator-activated receptors; ORT, object recognition test; s.c., subcutaneous; i.p., intraperitoneal; i.h., hippocampal; LTP, long-term potentiation; GSK3β, glycogen synthase kinase 3β; PI3K/Akt, phosphatidylinositol 3 kinase/protein kinase B; JNK, c-Jun-N-terminal kinases; cAMP, cyclic adenosine monophosphate; i.c.v., intracerebroventricular; i.g., intra-gastric; i.n., intranasal; NLRP2, nod-like receptor family pyrin domain containing 2; PPARγ, peroxisome proliferator-activated receptor gamma; mTOR, mammalian target of rapamycin; ROS, reactive oxygen species. “↑” for increase, “↓” for decrease.

The GLP-1 RAs involved in these articles include five types: liraglutide (*n* = 13), exenatide (*n* = 10), lixisenatide (*n* = 1), (Val8) GLP-1 (*n* = 1), and geniposide (*n* = 1). Liraglutide is based on the natural GLP-1 sequence and has 97% homology to human GLP-1, the main difference being the replacement of lysine with arginine at position 34 (Malm-Erjefält et al., 2010). It is a long-acting GLP-1 RAs, with a half-life of up to 13 h (Bain, 2014). Liraglutide was approved by the FDA in 2010. Currently, it is primarily administered through subcutaneous injection, and data indicates that the bioavailability of liraglutide reaches 55% after subcutaneous injection. Exenatide, a synthetic 39 amino acid peptide, is the first GLP-1 RAs approved for clinical use by the FDA in 2005 (Müller et al., 2019). Exenatide shares 53% homology with human GLP-1, and it can protect against DPP-IV cleavage and is resistant to DPP-IV inactivation (Diz-Chaves et al., 2022a). Lixisenatide is an analogue of exenatide and has comparable pharmacokinetic properties (Finan et al., 2015). Like exenatide, lixisenatide is also a short-acting GLP-1 RAs with a half-life of approximately 3 h. Adlyxin, which is a drug derived from lixisenatide, was approved by the FDA in 2016. (Val8) GLP-1 is a human GLP-1 analogue formed by substituting a Val residue for Ala at the N-terminal 8-position (Lennox et al., 2013). (Val8) GLP-1 has been shown to be resistant to cleavage by DPP-IV, thereby extending the half-life (Green et al., 2006). Geniposide, an iridoid glycoside present in the fruit of Gardenia jasminoides, is the main component of the traditional Chinese medicine Gardenia jasminoides (Hou et al., 2008). Recent studies have shown that geniposide can act as a GLP-1 RAs and induce various cellular signaling pathways by activating GLP-1 receptors (Liu et al., 2006; Gao et al., 2014).

Four animal models of AD were used in the article: transgenic animals (*n* = 14), chemical induction including injections of Aβ (*n* = 7), streptozotocin (STZ) (*n* = 4), and okadaic acid (OA) (*n* = 1). The characteristics of the following related models are summarized so as to have a better understanding of the underlying role of these animal models in the AD pathological mechanism unveiling and in their application for therapeutic trials.

(1) Transgenic animal models include Tg2576 mice, APP/PS1 mice, APPswe/PS1DE9, APPSwe/PSEN1 (*A246E*), 5×FAD mice, and 3×Tg mice. Tg2576 mice overexpress the Swedish mutation APP^*K670N/M671L*^ on the APP gene. This mutation leads to elevated levels of Aβ, which ultimately causes plaque deposition. In addition, the mice displayed oxidative stress injury and behavioral cognitive impairment. However, Tg2576 mice have no typical tau pathological manifestations and do not show neurofibrillary tangles (NFTs) (Irizarry et al., 1997). Both APP/PS1 mice, APPswe/PS1DE9 and APPSwe/PSEN1 (*A246E*) mice contain transgenes with the Swedish mutations in APP and PSEN1. These mice will develop amyloid deposits in the neocortex at around six weeks and a phosphorylated tau (p-Tau) neuritis process can be observed around the plaques, but without mature tangles and no typical tau pathological changes (Radde et al., 2006). 5×FAD mice express five AD-related mutations in the APP, such as the Swedish (APP^*K670N/M671L*^), Florida (APP^*I716V*^), and London (APP^*V717I*^) mutations, and PSEN1 genes, like the PS1^*M146L*^ and PS1^*L286V*^ mutations. It showed that 5×FAD mice only showed cognitive impairment associated with Aβ levels and did not show alterations in Tau pathology (Richard et al., 2015). 3×Tg mice include three mutations related to familial AD (APP *Swedish*, PSEN1 *M146V*, and MAPT *P301L*). These mice exhibited behavioral deficits in learning and memory abilities. Pathologically, they show progressive age-related changes, including tau protein hyperphosphorylation and Aβ pathology, with plaque and tangle formation. In addition, there is also present synaptic dysfunction and adenosine triphosphate (ATP) deficiency (Oddo et al., 2003). Transgenic mice are currently the most widely utilized animal models in preclinical studies of AD and have the advantage of exhibiting familial AD-related manifestations (Puzzo et al., 2015). A major limitation of Tg2576 mice and double transgenic mice (APP/PS1 mice, APPSwe/PSEN1 (*A246E*), and 5×FAD mice is the absence of typical tau pathology. Although p-Tau is observed in some models, it does not develop into NFTs. Although both amyloid plaques and NFTs were present in 3×Tg mice, the study showed that these pathologies developed late, and were not observed until the mice aged (Drummond and Wisniewski, 2017).
(2) Chemically induced models include injections of Aβ, STZ and OA. The Aβ infusion model induces neurotoxicity by injecting Aβ peptides with manifested Aβ deposition, inflammatory response and cognitive impairment. It is mainly used in the study of Aβ pathology. Since injection of Aβ will cause acute toxicity, this model cannot recapture the progressive onset of AD, moreover the concentration of injection sites may lead to uneven distribution of Aβ deposition sites and damage the brain’s surrounding tissue (Jia-Yue et al., 2019). The STZ infusion model, first proposed by Professor Sigfried Hoyer, is considered to be useful in the study of sporadic AD (Hoyer et al., 1994; Salkovic-Petrisic et al., 2013). He suggested that the major biochemical perturbation in sporadic AD involves controlling cerebral glucose metabolism, which follows the failure of brain insulin receptor signaling (Hoyer et al., 1993). Intracerebroventricular (i.c.v.) injection of STZ can disrupt the control level of glucose and energy metabolism, resulting in decreased brain glucose utilization, disturbed brain metabolism and cognitive dysfunction (Nitsch and Hoyer, 1991; Duelli et al., 1994; Grieb et al., 2004; Stranahan et al., 2008). In addition to impaired brain metabolism, the STZ model exhibited tau hyperphosphorylation, amyloid deposition, oxidative stress, and neuroinflammation (Salkovic-Petrisic et al., 2009; Tiwari et al., 2009; Saxena et al., 2011; Chen et al., 2013; Correia et al., 2013). The high mortality rate of animals after STZ injection is a limitation of this model (Lester-Coll et al., 2006). OA is a selective inhibitor of protein phosphatases that causes neuronal cell death (Kamat et al., 2014). Animals injected with OA will develop AD-like pathology, including tau protein hyperphosphorylation, amyloid deposition, memory impairment, synapse loss, etc., (Arias et al., 1998; Yoon et al., 2006; Kamat et al., 2013).

In summary, these animal models have their own advantages and limitations. Compared to the various models, their phenotypic similarities are more noteworthy (Esquerda-Canals et al., 2017). Therefore, the selection of appropriate animal strains and modeling methods deserve attention when conducting further experiments.

### 3.2 Behavioral test analysis

**FIGURE 3:**
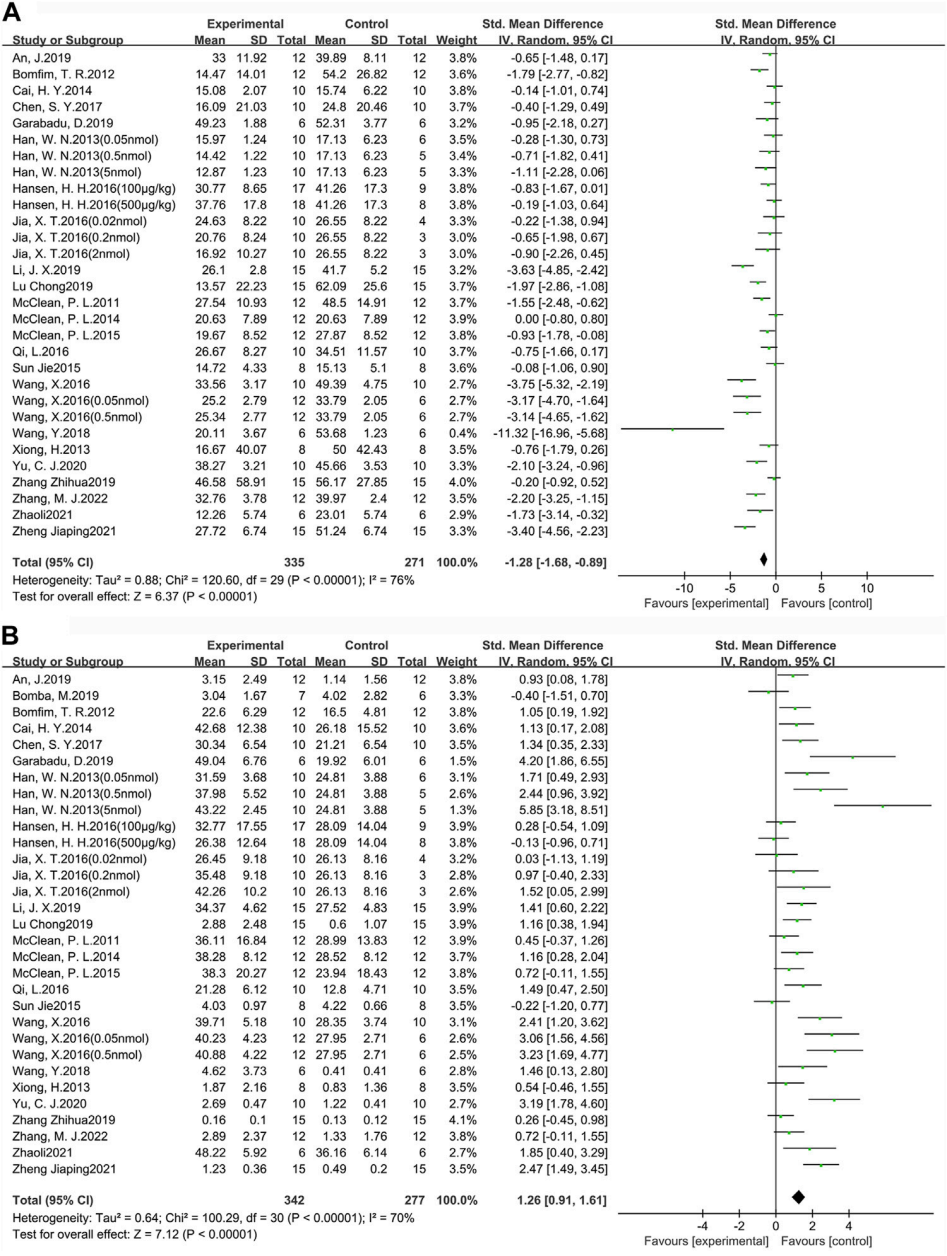
Forest plot: effect of GLP-1 RAs on learning and memory abilities. **(A)** represents the result of the learning test, which was determined by measuring the escape latency; **(B)** represents the result of the memory test, which was determined by measuring the target quadrant dwell time and the percentage of time in the target quadrant dwell.

MWM is one of the most widely used tasks for researching the spatial learning and memory abilities of AD animal models (Pantoni and Anagnostaras, 2019). In this study, escape latency was chosen as an indicator to evaluate learning ability, while the target quadrant dwell time and the percentage of time in the target quadrant dwell were used as indicators to assess memory ability. A total of 24 studies (30 groups) with 606 animals (experimental group *n* = 335, control group n = 271) were used to assess learning ability (Figure 3A). In addition, there were 25 studies (31 groups) with 619 animals (experimental group *n* = 342, control group n = 277) used to assess memory ability (Figure 3B). Compared to the control group, learning ability (SMD = −1.28, 95% CI = −1.68 to −0.89, *p* < 0.01, *I* ^2^ = 76%) and memory ability (SMD = 1.26, 95% CI = 0.91 to 1.61, *p* < 0.01, *I* ^2^ = 70%) were significantly improved in AD animals after GLP-1 RAs treatment. Separate subgroup comparisons of learning and memory abilities were performed according to drugs and modeling methods, which were used to reduce heterogeneity and further analyze the effect of GLP-1 RAs. Due to the fact that only one study is available for each of lixisenatide, (Val8) GLP-1, and geniposide, subgroup analysis cannot be performed on them. Therefore, this study only conducts subgroup analysis on the studies involving liraglutide and exenatide.

#### 3.2.1 Learning ability

**FIGURE 4:**
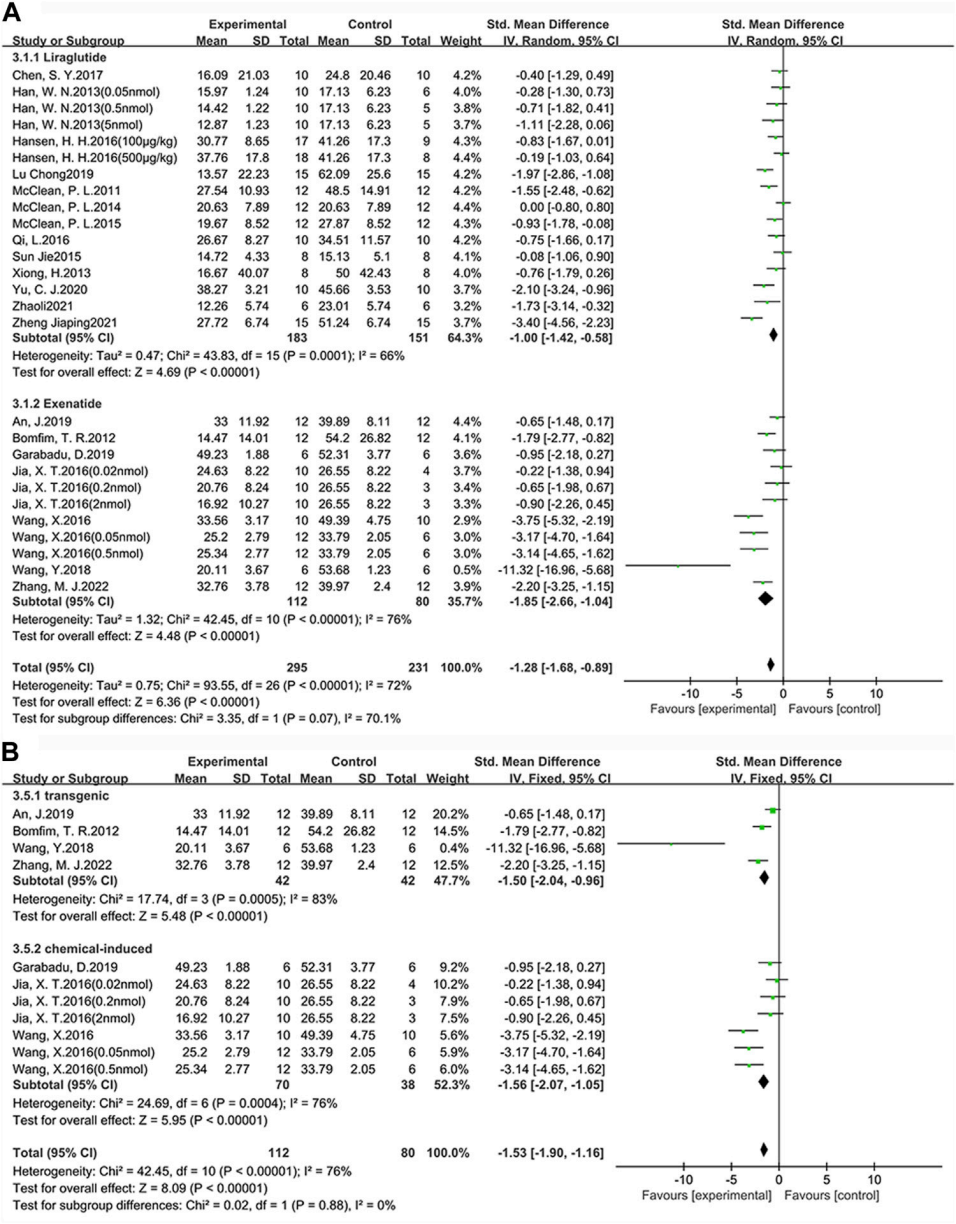
Forest plot depicting subgroup analyses to determine the effects of different factors on learning ability. **(A)** Drugs; **(B)** Animal models treated with exenatide.

Among the 24 studies (30 groups) in which learning ability was tested, 16 groups chose liraglutide, while 11 groups chose exenatide (Figure 4A). As for the ability of GLP-1 RAs to correct learning deficits, the exenatide group (SMD = −1.85, 95% CI = −2.66 to −1.04, *p* < 0.01, *I* ^2^ = 76%) and the liraglutide group (SMD = −1.00, 95% CI = −1.42 to −0.58, *p* < 0.01, *I* ^2^ = 66%) were respectively compared with the control group, suggesting that exenatide group displayed more significant improvements.

In the exenatide group, four groups selected the transgenic animal model (SMD = −1.50, 95% CI = −2.04 to −0.96, *p* < 0.01, *I* ^2^ = 83%), and seven groups selected the chemical-induced animal model (SMD = −1.56, 95% CI = −2.07 to −1.05, *p* < 0.01, *I* ^2^ = 76%) (Figure 4B). The result showed that chemical-induced animal model group displayed more significant amelioration in learning ability compared to the control group.

#### 3.2.2 Memory ability

**FIGURE 5:**
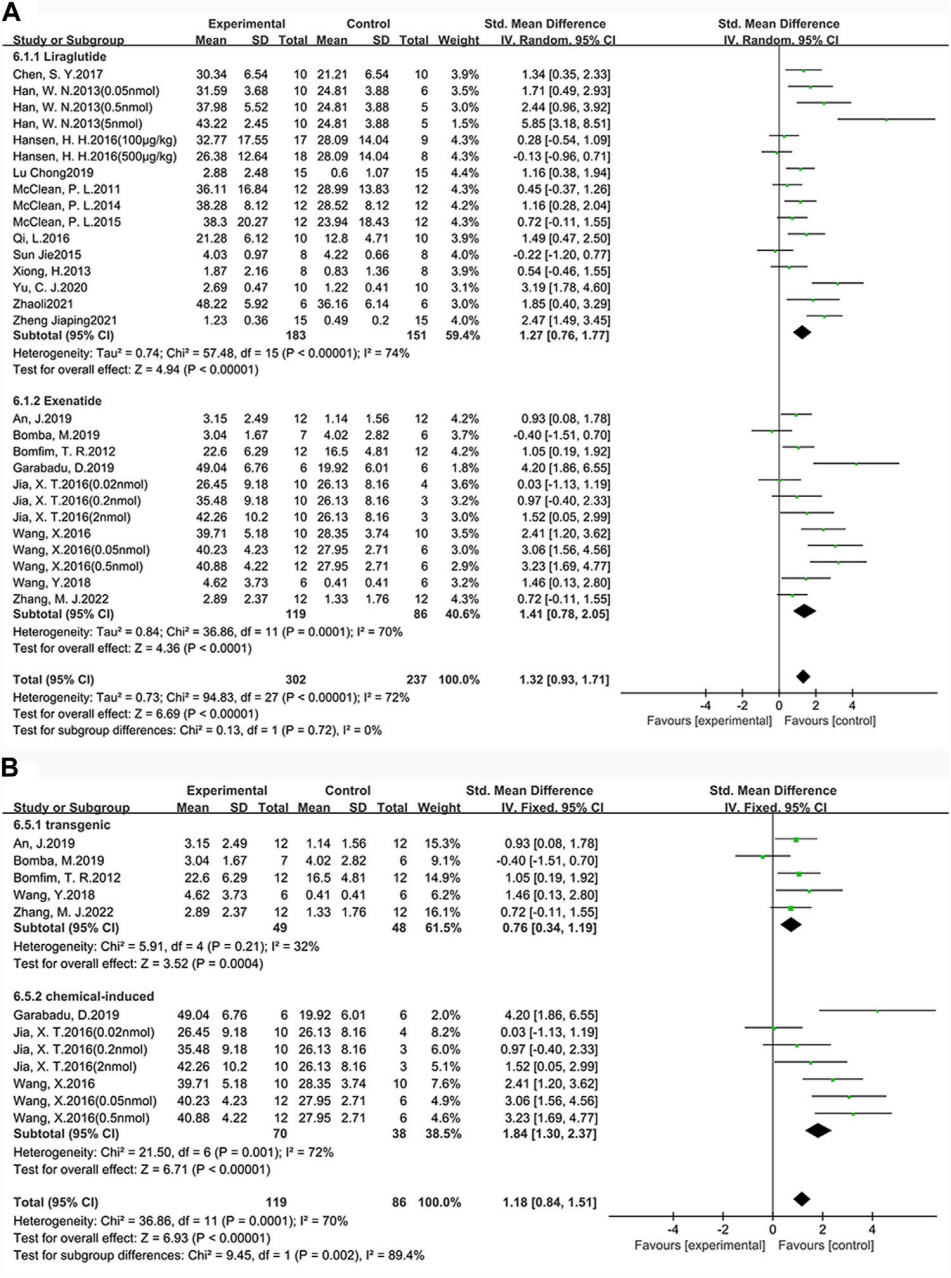
Forest plot depicting subgroup analyses to determine the effects of different factors on memory ability. **(A)** Drugs; **(B)** Animal models treated with exenatide.

Among the 25 studies (31 groups) in which memory ability was tested, 16 groups chose liraglutide, while 12 groups chose exenatide (Figure 5A). As for the ability of GLP-1 RAs to correct memory deficits, the exenatide group (SMD = 1.41, 95% CI = 0.78 to 2.05, *p* < 0.01, *I* ^2^ = 70%) and the liraglutide group (SMD = 1.27, 95% CI = 0.76 to 1.77, *p* < 0.01, *I* ^2^ = 74%) were respectively compared with the control group, suggesting that exenatide group displayed more significant improvements.

In the exenatide group, five groups selected the transgenic animal model (SMD = 0.76, 95% CI = 0.34 to 1.19, *p* < 0.01, *I* ^2^ = 32%) and seven groups selected the chemical-induced animal model (SMD = 1.84, 95% CI = 1.30 to 2.37, *p* < 0.01, *I* ^2^ = 72%) (Figure 5B). The result showed that chemical-induced animal model group displayed more significant amelioration in memory ability compared to the control group.

This indicates that, in comparison to liraglutide, exenatide shows a greater improvement in learning and memory abilities in AD animals. Furthermore, concerning the modeling methods, exenatide demonstrates a better ability to correct cognitive deficits in the chemically induced animal model.

### 3.3 Neuropathological features

Plaques and NFTs are two important pathological features in the diagnosis of AD (Scheltens et al., 2021). Aβ plaques are obtained through the cleavage of amyloid precursor proteins (APP) by β-secretase and γ-secretase (Calsolaro and Edison, 2016). Hyperphosphorylation of tau protein is an early sign of NFT pathogenesis (Kannanayakal et al., 2006). Tau proteins are microtubule neuronal proteins involved in the polymerization and stabilization of microtubule assembly to maintain the integrity of the cytoskeleton (Khan et al., 2020). Both Aβ and tau can aggregate and cause synaptic damage as well as neuronal cell death. In this study, Aβ deposition and p-Tau were selected as secondary indicators to investigate the therapeutic effects of GLP-1 RAs in AD animals.

#### 3.3.1 Aβ deposition

**FIGURE 6:**
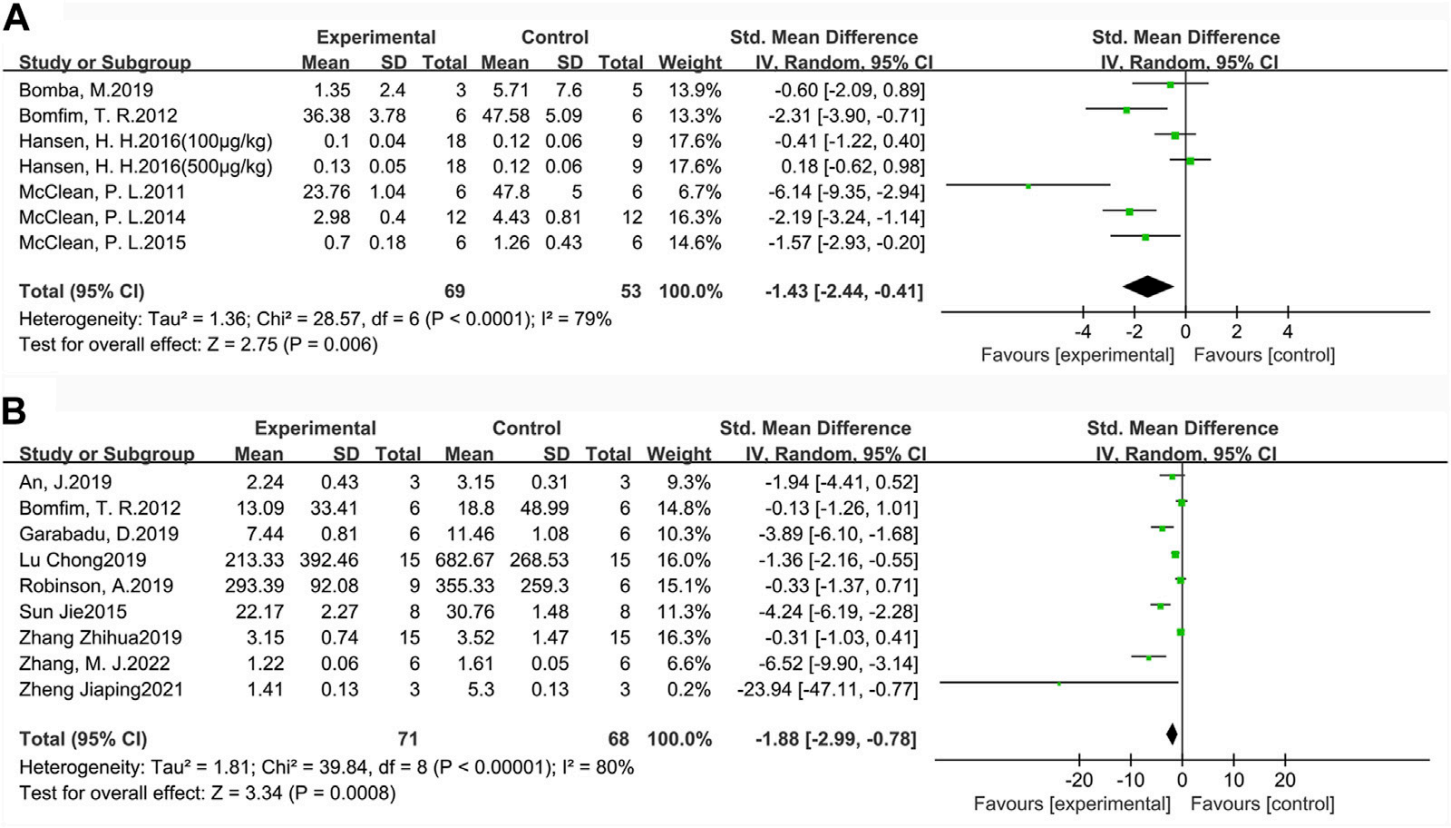
Forest plot: effect of GLP-1 RAs on **(A)** Aβ plaque burden, **(B)** Aβ_1-42_ deposition.

For a more comprehensive assessment of Aβ pathology in the brains of AD animals, we selected the indicators of Aβ plaque burden (Figure 6A) and Aβ_1-42_ deposition (Figure 6B) to analyze the effect of GLP-1 RAs. Aβ plaque burden (SMD = −1.43, 95% CI = −2.44 to −0.41, *p* < 0.01, *I* ^2^ = 79%) was tested by immunohistochemistry in six studies (seven groups), involving 122 animals (experimental group n = 69, control group n = 53). Aβ_1-42_ deposition (SMD = −1.88, 95 %CI = −2.99 to −0.78, *p* < 0.01, *I* ^2^ = 80%) was detected by ELISA in nine studies involving 139 animals (experimental group *n* = 71, control group *n* = 68). The results indicated that GLP-1 RAs treatment could effectively reduce Aβ plaque burden and Aβ_1-42_ in the brains of AD animals to some extent.

#### 3.3.2 Phosphorylated tau

**FIGURE 7:**
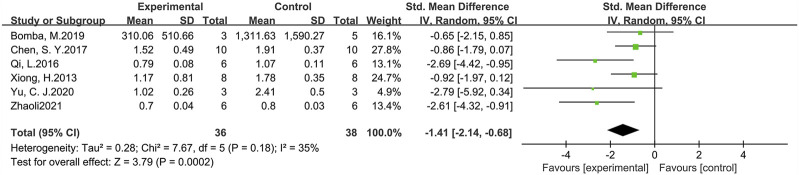
Forest plot: effect of GLP-1 RAs on p-Tau levels.

p-Tau levels were measured in six studies involving 74 animals (experimental group n = 36, control group *n* = 38) (Figure 7). The results suggested that GLP-1 RAs were effective in reducing p-Tau levels (SMD = −1.41, 95% CI = −2.14 to −0.68, *p* < 0.01, *I* ^2^ = 35%) in animal models of AD.

### 3.4 Administration method

**FIGURE 8:**
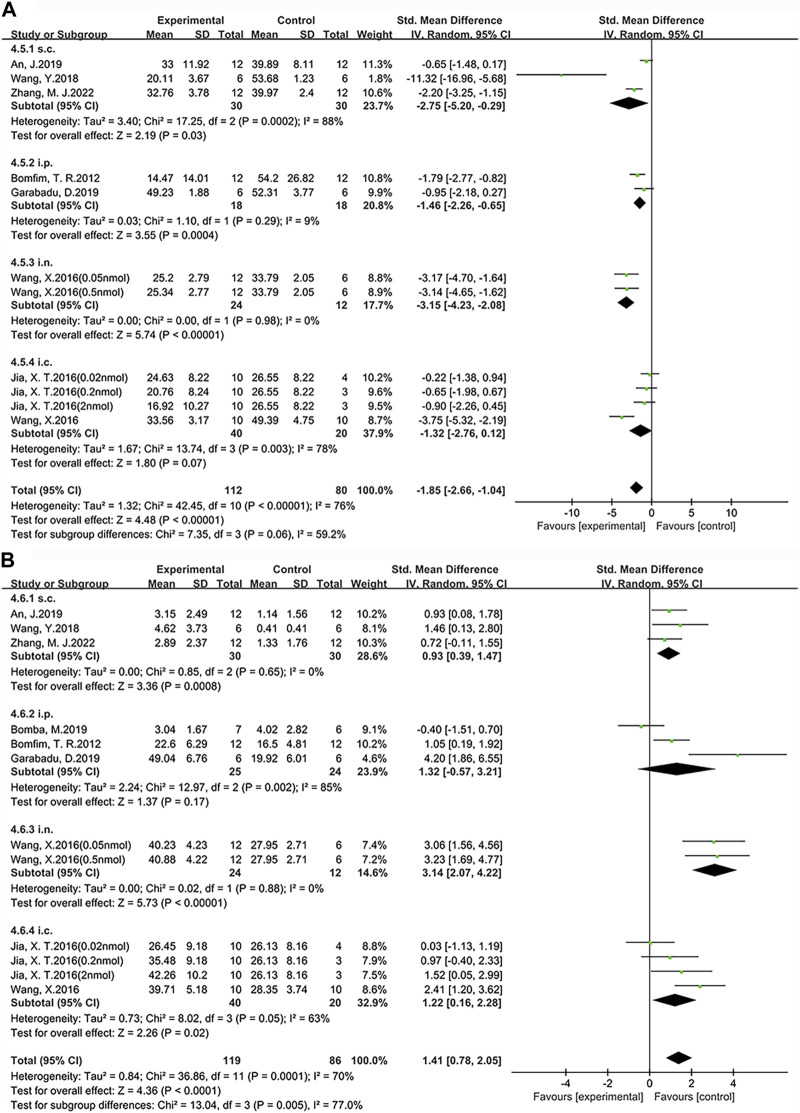
Forest plot: the effects of different administration method of exenatide on the improvement of cognitive function. **(A)** Learning ability **(B)** Memory ability.

Based on the above research findings, this study investigates the therapeutic effects of different doses and administration methods of exenatide on improving learning (Figure 8A) and memory impairments (Figure 8B) in AD. The doses were firstly standardized to μg/kg and the doses given by rats were converted to mice doses by means of the formula (Janhavi et al., 2022), followed by a comparison. The administration methods of exenatide involve a total of five approaches, including intranasal (i.n.) administration, subcutaneous (s.c.) administration, intraperitoneal (i.p.) administration, hippocampal (i.h.) administration, and i.c.v. In this study, i.p. and i.c.v. administrations are categorized collectively as intracerebral (i.c.) injections. The analysis will be conducted on these four administration methods.

According to our findings, among the four administration routes compared with the control group, i.n. administration (SMD = −3.15, 95% CI = −4.23 to −2.08, *p* < 0.01, *I* ^2^ = 0%) demonstrated a significant improvement in learning ability. Specifically, the other statistical results are as follows: s.c. administration (SMD = −2.75, 95% CI = −5.20 to −0.29, *p* < 0.05, *I* ^2^ = 88%), i.p. administration (SMD = −1.46, 95% CI = −2.26 to −0.65, *p* < 0.01, *I* ^2^ = 9%) and i.c. administration (SMD = −1.32, 95% CI = −2.76 to 0.12, *p* = 0.07, *I* ^2^ = 78%). Additionally, among the four administration routes, i.n. administration (SMD = 3.14, 95% CI = 2.07 to 4.22, *p* < 0.01, *I* ^2^ = 0%) demonstrated a significant improvement in memory ability compared to the control group. Specifically, the other statistical results are as follows: i.p. administration (SMD = 1.32, 95% CI = −0.57 to 3.21, *p* = 0.17, *I* ^2^ = 85%), i.c. administration (SMD = 1.22, 95% CI = 0.16 to 2.28, *p* < 0.05, *I* ^2^ = 63%) and s.c. administration (SMD = 0.93, 95% CI = 0.39 to 1.47, *p* < 0.01, *I* ^2^ = 0%). Therefore, we conclude that single i.n. administration of exenatide demonstrates advantages in improving learning and memory abilities in AD animals, with dosages ranging from 10.47 μg/kg to 104.68 μg/kg.

## 4 Discussion

GLP-1 RAs can exert neuroprotective effects by binding to GLP-1 receptors and activating downstream signaling, thus having therapeutic potential for AD (Cantini et al., 2016; Hölscher, 2020). In GLP-1 RAs treatment, several mechanisms have been indicated to have beneficial effects on the brain, including reduction of neuroinflammation, enhancement of synaptic transmission, and reduction of learning deficits (During et al., 2003; Salcedo et al., 2012). In addition, GLP-1 RAs can promote the proliferation of neural stem cells in rodents (Bertilsson et al., 2008; Hamilton et al., 2011). The current studies on the therapeutic use of GLP-1 RAs for AD are mainly in the preclinical stage. The reliability of the conclusions can be improved by analyzing and integrating existing experiments. In this study, rodent models were selected to explore the ameliorating effects and mechanisms of action of GLP-1 RAs on AD.

In this study, MWM was selected as the main indicator to evaluate the effects of GLP-1 RAs for ameliorating the cognitive function of AD animals by escape latency, and the target quadrant dwell time or the percentage of time in the target quadrant dwell, respectively. The results of the meta-analysis indicated that GLP-1 RAs could effectively increase the cognitive function of AD animals. According to subgroup analysis, in different modeling methods, GLP-1 RAs could ameliorate cognitive performance more markedly in chemically induced model animals; in terms of animal strains, GLP-1 RAs showed more significant improvement in rats for cognitive impairment. For pathological features, Aβ deposition and p-Tau protein were selected. The results showed that GLP-1 RAs reduced Aβ deposition and tau protein hyperphosphorylation in the brains of AD animals.

The incidence and clinical manifestations of AD show gender differences. Investigation reveals that women suffer from AD at a much higher rate than men, and female AD patients have more prominent pathologic manifestations, such as greater brain atrophy and cognitive impairment (Guo et al., 2023). This disparity seems to be influenced by various factors, with some studies suggesting that the association between APOE4 and AD is more prominent in females (Corder et al., 2004; Ungar et al., 2014). A network analysis showed that Lipoprotein receptor-related protein 10 (LRP10), with high sex differential expression, is a critical regulatory factor in female AD. And the finding was successfully validated by the EFAD mouse model (Guo et al., 2023). LRP10 is considered to influence cognitive function and AD pathology in a manner that is specific to gender and APOE genotype. LRP10 is thought to reduce Aβ production by influencing the maturation of APP(Brodeur et al., 2012). In studies involving AD patients, it was observed that the levels of LRP10 protein in the brains of AD patients were significantly lower compared to the control group, with a more pronounced reduction observed in females (Brodeur et al., 2012). In female AD, reduced LRP10 protein expression leads to impaired APP transport and increased Aβ. Furthermore, the reduction of LRP10 also results in a decrease in microglia clearance of Aβ (Brodeur et al., 2012; Guo et al., 2023). Moreover, a recent study unveiled that the propagation of NFTs occurs at a higher pace in the brains of females compared to males. This accelerated spread could potentially be attributed to the unique network structures of brain regions influenced by tau (Shokouhi et al., 2020). Meanwhile, it has been suggested by studies that intact mitochondrial function could more effectively shield females from the toxic effects of Aβ. This might be attributed to estrogen-mediated ROS inhibition, which, when estrogen levels decline after menopause, could result in greater susceptibility of females to mitochondrial dysfunction (Viña and Lloret, 2010).

Furthermore, research on T2DM has revealed that estrogen also exerts an influence on insulin resistance and glucose metabolism (Mauvais-Jarvis et al., 2013; Root-Bernstein et al., 2014). Moreover, influenced by estrogen, central stimulation of GLP-1 RAs has been found to be more sensitive in females (Richard et al., 2016). Therefore, whether there is a differential therapeutic effect of GLP-1 RAs on AD warrants further attention and in-depth investigation.

Despite abundant evidence supporting the gender-specificity of AD, only a few experiments have focused on gender differences, leading to an underestimation of the importance of gender (Guo et al., 2022). One of the studies included in our research did not differentiate animal sex. Furthermore, the selection of animal gender in previous studies has been uneven. Our research found that most of the current research has predominantly chosen male animals as subjects, which may be an attempt to control for variables and prevent sex differences from having an effect on AD pathology. However, as the deepening of AD gender research, the use of female animals as AD models is gradually gaining attention. In our research, there has one study selected female mice as subjects, and more AD experiments specifically targeting female animals should be carried out in the future.

A comprehensive analysis of the improvement of learning and memory abilities by drugs. Based on our conclusions, exenatide administered intranasally at doses ranging from 10.47 to 104.68 μg/kg exhibited a more significant enhancement of cognitive function in mice. After converting the mouse doses to human doses, we recommend a human dose range of exenatide between 59.42 and 594.13 μg/70 kg. The i.n. administration facilitates direct drug delivery to the brain, enabling faster access to the CNS. Moreover, it effectively circumvents the first-pass effect through the gastrointestinal tract and liver, thereby enhancing the drug’s bioavailability (Shehata et al., 2023). While our study highlights the pronounced advantages of intranasal administration, it is noteworthy that current clinical practice primarily employs subcutaneous injection for exenatide, which boasts benefits such as simplicity, safety, and rapid absorption. Therefore, the doses and cycles given in this study are only preliminary recommendations, and future optimization and exploration of the results based on bioavailability are necessary to find the appropriate cycles and doses for clinical application.

### 4.1 Potential mechanisms

Drawing from the compilation of 26 studies, it becomes evident that GLP-1 RAs play a therapeutic role in AD by collaboratively ameliorating diverse pathological manifestations associated with AD.

The connection between T2DM and AD may potentially stem from abnormalities in insulin signaling and insulin resistance mechanisms (Grieco et al., 2019). A large number of studies have shown that insulin signaling is impaired in the brains of AD patients and AD animals (Bomfim et al., 2012b; Talbot et al., 2012). The insulin signaling process consists of insulin binding to the insulin receptor (IR), enabling IR activation and autophosphorylation (Hubbard, 1997; Hubbard, 2013). Activation of tyrosine kinases on IR can aggregate and phosphorylate insulin receptor substrate (IRS), which binds and activates downstream pathways after phosphorylation (Nguyen et al., 2020). Phosphatidylinositol 3 kinase/protein kinase B (PI3K/AKT) and Mitogen-activated protein kinase (MAPK) are the two main signaling pathways (Griffith et al., 2018). PI3K/AKT signaling promotes energy metabolism, protein and lipid synthesis, and glucose transport (Kleinridders et al., 2014). It has also been found to ameliorate cognitive function by improving memory consolidation and modulating synaptic plasticity (Chiang et al., 2010). Glycogen synthase kinase 3β (GSK3β) is a protein kinase that is considered to accelerate tau protein phosphorylation and the formation of NFTs (Zheng and Wang, 2021). Tau contains more than 80 potential phosphorylation sites located at serine, tyrosine and threonine (Noble et al., 2013), and phosphorylation of these sites is regulated by protein kinases and protein phosphatases. When GSK3β is activated, it can increase the phosphorylation of Tau proteins (Yu et al., 2020). When tau is hyperphosphorylated, it decreases the affinity for microtubules, and leads to the formation of NFTs (Lee et al., 2001; Reddy and Reddy, 2011). Moreover, the activation of GSK3β has been observed to elevate Aβ levels and promote oxidative stress, which ultimately contributes to cognitive deterioration. When PI3K/AKT is activated, GSK3β activity can be inhibited to alleviate AD symptoms.

MAPK is another major insulin signaling pathway, consisting mainly of ERK, c-Jun-N-terminal kinases (JNKs) and p38 (Sędzikowska and Szablewski, 2021). In clinical practice, it has been found that patients with AD exhibit excessive expression of P38 in the brain (Gourmaud et al., 2015). The overexpression of P38 increases the risk of tau protein phosphorylation and promotes abnormal processing of APP (Ali et al., 2017). In AD mouse models, it was found that JNK is excessively activated and disrupts synaptic plasticity before cognitive decline occurs (Sclip et al., 2014). At the same time, when JNK is activated, it inhibits tyrosine phosphorylation of IRS-1, thereby disrupting insulin signaling. Aβ oligomers in the brain promote activation of the JNK signaling pathway, resulting in reduced IRS, inhibition of PI3K/AKT, and increased GSK3β activity, ultimately leading to an increase in Aβ, p-Tau and a decline in cognitive function. GLP-1 RAs can activate G proteins by binding to GLP-1 R. This activation will lead to an increase in PI3K levels, which restores insulin signaling and inhibits GSK3β activity (Hölscher and Li, 2010). As a result, Aβ and p-Tau levels are reduced and cognitive function is improved. Studies in AD rodent models have found that AKT activation and GSK3β inhibition were observed in the brain following the administration of GLP-1 RAs (Cai et al., 2014; Qi et al., 2016; Wang et al., 2018; Li J. X. et al., 2019; Garabadu and Verma, 2019; Robinson et al., 2019; Zheng, 2021). In addition, GLP-1 RAs were found to reduce the phosphorylation levels of JNK and IRS-1 serine in animals, thereby restoring insulin signaling (Bomfim et al., 2012a; Xiong et al., 2013; Chen et al., 2017; Li J. X. et al., 2019). In addition to reducing Aβ levels by restoring insulin signaling channels GLP-1 RAs can also directly reduce Aβ production by reducing abnormal cleavage of APP. Aβ is a toxic fragment produced when APP is abnormally cleaved by β-secretase and γ-secretase, and these fragments accumulate and precipitate to form plaques (Götz and Ittner, 2008; Woodruff et al., 2013). Aβ aggregation could lead to neuronal loss and increase the risk of oxidative stress, mitochondrial dysfunction and inflammation (Bateman et al., 2022). GLP-1 RAs have been found to reduce the generation of Aβ by inhibiting the level of BACE1 and increasing the level of α-secretase, thus exerting a therapeutic effect on AD (McClean et al., 2015; Wang et al., 2016a; Wang et al., 2016b).

Impaired insulin signaling and Aβ accumulation are factors that promote the occurrence of insulin resistance (Pelle et al., 2023). Insulin resistance refers to decreased sensitivity of tissues to insulin, and neuronal insulin resistance is considered a key feature of AD (Jantrapirom et al., 2020). Insulin plays a crucial role in various brain functions, including cognitive function and synaptic plasticity. Substantial evidence indicates a correlation between reduced insulin sensitivity and the development of AD (Dou et al., 2005; Rivera et al., 2005; Chiu et al., 2008; Hölscher and Li, 2010). The impact of insulin resistance on AD pathology involves multiple aspects. Insulin resistance can lower the activity of insulin-degrading enzyme (IDE) in the brain (Jantrapirom et al., 2020). IDE is responsible for clearing insulin and Aβ. When IDE activity is reduced, insufficient Aβ clearance leads to its accumulation. Simultaneously, insulin resistance can enhance BACE-1 activity, thereby increasing Aβ production. Aβ accumulation further elevates serine phosphorylation of IRS-1, disrupting insulin signaling, releasing inflammatory factors, and exacerbating insulin resistance (Kurochkin et al., 2018). Furthermore, insulin resistance has been found to heighten oxidative stress, manifested by increased reactive oxygen species (ROS) levels, consequently promoting the secretion of inflammatory factors and mitochondrial dysfunction (Boccardi et al., 2019). Meanwhile, states of insulin resistance influence PI3K-mediated vasodilation. This could contribute to a reduction in nutrient supply to the brain (Lynn et al., 2022).

It has been observed that patients with AD have reduced brain glucose metabolism rates, specifically within areas linked to memory consolidation and cognitive acquisition (Reiman et al., 2004; Mosconi et al., 2008; Mistur et al., 2009). Glucose primarily generates ATP to supply energy for various neuronal activities in the brain. Decreased glucose metabolism leads to insufficient ATP production, affecting a cascade of physiological functions. This manifests as impaired Ca^2+^ signaling and depolarization (Alzheimer’s Association Calcium Hypothesis Workgroup, 2017), resulting in endoplasmic reticulum (ER) stress, mitochondrial dysfunction, and increased ROS generation (Hachinski et al., 2019). Concurrently, compromised brain glucose metabolism compels the brain to seek alternative sources of energy. For instance, the brain activates lipid-digesting enzymes to produce ATP (Klosinski et al., 2015). However, this process triggers the generation of hemolytic phospholipids, inducing inflammation and oxidative stress (Schönfeld and Reiser, 2013; Sato et al., 2016). GLP-1 RAs have been found to enhance brain glucose metabolism by regulating glucose transporter proteins GLUT1 and GLUT3 through cyclic adenosine monophosphate (cAMP) activation (Yassine et al., 2022). Research indicates that concentrations of GLUT1 and GLUT3 are reduced in the neocortex and hippocampus of AD patients, suggesting this may be one of the primary reasons for decreased glucose uptake (Simpson et al., 1994; Harr et al., 1995). Therefore, GLP-1 RAs hold promise in improving glucose metabolism by restoring GLUT1 and GLUT3 concentrations, potentially offering a treatment avenue for AD. Autopsy reports of AD patients have shown increased brain glucose levels, even in the absence of coexisting T2DM(An et al., 2018). Hyperglycemia affects the accumulation of advanced glycation end products (AGEs). The binding of AGEs to the receptor for advanced glycation end products (RAGE) triggers a series of pathological reactions, including disruption of insulin receptor binding to IRS-1, promoting pro-inflammatory cytokine production, increasing ROS generation, and leading to synaptic dysfunction (Lynn et al., 2022). AGEs can also induce glycation of Aβ and tau proteins, resulting in the aggregation of Aβ into plaques and the entanglement of neurofibrillary tangles (Iannuzzi et al., 2014; Vlassara and Uribarri, 2014). Simultaneously, RAGE is believed to be involved in Aβ-induced ER stress. ER stress plays a complex role in neuroprotection, amyloid-beta deposition, and synaptic function regulation in AD. The endoplasmic reticulum is a fundamental organelle responsible for synthesizing essential biomolecules such as proteins, lipids, and carbohydrates within cells. When cells are exposed to stress stimuli, improper and incomplete folding of proteins and disrupted calcium ion balance can lead to ER stress. ER stress has been shown to be involved in various metabolic disorders and neurodegenerative diseases. Upon activation of ER stress, JNK is activated, leading to serine phosphorylation of IRS1, thus compromising insulin signaling (Urano et al., 2000; Nakatani et al., 2005; Kawasaki et al., 2012). Insulin resistance may also exacerbate ER stress in AD. GLP-1 RAs have been demonstrated to intervene in ER stress and protect against neurodegenerative diseases, suggesting that they hold promise as potential therapeutic agents for AD by modulating ER stress (Cheng et al., 2022).

Oxidative stress is caused by a disruption in the body’s homeostasis between oxidative and antioxidant action, mainly manifested as excessive production or insufficient clearance of ROS and reactive nitrogen species (Reddy et al., 2009). Oxidative stress is one of the pathogenic mechanisms of AD, and an increase in ROS can be observed in AD patients (Ahmad et al., 2017). Aβ deposits exist in the AD brain, and these Aβ can penetrate the mitochondrial membrane and bind to mitochondria, thereby disrupting mitochondrial oxidative phosphorylation, increasing ROS levels, and ultimately leading to mitochondrial dysfunction and synaptic degeneration (An et al., 2019). Excessive ROS generation can produce various AGEs and advanced lipid end products (ALE) (Sayre et al., 2006). AGEs and ALEs can interact with the RAGE, inducing a pro-inflammatory cytokine response. Meanwhile, RAGE can also interact with Aβ, exacerbating neuroinflammation and causing a decline in learning ability (Harris et al., 2000). Oxidative stress can also increase the levels of p-Tau and Aβ by activating p38MAPK. In addition, increased oxidative stress may accelerate apoptosis, produce inflammatory mediators and damage neurons. Superoxide dismutase (SOD) and malondialdehyde (MDA) as important markers of oxidative stress are often used to assess the level of oxidative stress (Chen et al., 2020). Studies have shown that treatment with GLP-1 RAs could significantly increase the level of SOD and reduce the level of MDA in the AD rodents’ brains (An et al., 2019; Li J. X. et al., 2019). Meanwhile, GLP-1 RAs can also reduce oxidative stress by increasing the level of reduced glutathione (GSH), an important intracellular antioxidant in the body that counteracts the damage caused by antioxidants to cells, and reducing ROS levels (Zheng, 2021; Zhang et al., 2022). Oxidative stress and inflammation interact, which contributes to the pathology of AD (Tadokoro et al., 2020). The brain generates an inflammatory response primarily through the activation of microglia and astrocytes (Chakrabarti et al., 2015). Studies have found that the levels of pro-inflammatory cytokines are significantly increased in serum and brain tissue of AD patients, and activated microglia and astrocytes could be observed in the brain (Fillit et al., 1991; Strauss et al., 1992; Sastre et al., 2006). Not only can oxidative stress promote the production of pro-inflammatory factors that exacerbate inflammation, but microglia stimulated by Aβ can also release ROS, causing oxidative stress (Akiyama et al., 2000). Microglia and astrocytes are essential for maintaining brain homeostasis and supporting neuronal function (Jha et al., 2019). During the progression of AD, microglia will lose their homeostatic phenotype and be activated for a long time, manifested as glial proliferation and the secretion of pro-inflammatory factors, and these mediators will promote astrogliosis and intensify the inflammatory response (Diz-Chaves et al., 2022b). Activated microglia and astrocytes, in addition to producing direct neurotoxicity, also lead to increased Aβ deposition (Guo et al., 2002). At the same time, the accumulation of Aβ also promotes the secretion and release of pro-inflammatory cytokines, exacerbating the inflammatory response (Akiyama et al., 2000). GLP-1 RAs are considered to improve the inflammatory response in AD by increasing the level of brain-derived neurotrophic factor (BDNF) (Bomba et al., 2019). Meanwhile, GLP-1 RAs have also been found to inhibit the activation of microglia (McClean et al., 2011; 2015; McClean and Hölscher, 2014) and astrocytes (Matsuoka et al., 2019; Zheng, 2021; Zhang et al., 2022) as well as reduce the expression of pro-inflammatory factors such as tumor necrosis factor-α (TNF-α), interleukin 1β (IL-1β) and interleukin 18 (IL-18) (Zhang et al., 2022).

Mitochondrial dysfunction is also one of the pathological manifestations of AD. Mitochondria serve as the main energy production system within cells, and impairment of mitochondria can lead to neuronal dysfunction, resulting in cognitive impairment. Peroxisome proliferator-activated receptor c coactivator 1a (PGC-1a), an important factor in mitochondrial biogenesis, plays a crucial role in synaptic and CNS function. Research has shown that GLP-1 RAs can directly reduce p-Tau levels and restore mitochondrial function through modulation of the PGC-1a pathway (An et al., 2015). Meanwhile, GLP-1 RAs may also improve mitochondrial function by activating the extracellular signal-regulated kinase-Yes-associated protein signaling pathway and the CREB/adiponectin axis (Li J. et al., 2019; Xiong et al., 2020). Mitochondrial dysfunction leads to abnormalities in multiple biological processes, such as excessive production of ROS. These abnormalities subsequently result in synaptic degeneration of neurons, characterized by structural and functional alterations at the synapses (Tillement et al., 2011). Synaptic dysfunction is considered one of the main neuropathological changes that occur before clinical symptoms of AD appear (Fu and Ip, 2022). It found that synaptic loss can be observed in the mild cognitive impairment of AD early stage (Scheff et al., 2006). The basis of normal synaptic function is recognized as synaptic plasticity, and when synaptic plasticity is impaired, information transmission in the CNS is disrupted, leading to impaired cognitive function (Yu et al., 2022). It has been found that Aβ deposition can affect the levels of presynaptic markers and postsynaptic markers which disrupt synaptic plasticity (Bakota and Brandt, 2016; Forner et al., 2017). In addition, hyperphosphorylation of tau is another factor that leads to synaptic dysfunction (Wu et al., 2021). When p-Tau increases, it destroys microtubule structures, consequently disrupting microtubule-based cellular transport, and synaptic loss occurs when mitochondria are not efficiently transported to the synapse (Forner et al., 2017). Damage to synaptic structure and function can lead to symptom aggravation, such as impaired memory and difficulty moving, so AD symptoms could be ameliorated by restoring synaptic structure and function (John and Reddy, 2021). It was found that GLP-1 RAs intervention can alleviate synaptic structure and functional damage in AD rodent models, specifically manifested as an increase in levels of postsynaptic markers (postsynaptic density protein 95, PSD95) and presynaptic markers (synaptophysin, SYN), and significant enhancement of hippocampal long term potentiation (LTP) (McClean et al., 2011; McClean et al., 2015; Han et al., 2013; Cai et al., 2014; McClean and Hölscher, 2014; Wang et al., 2016a; Qi et al., 2016; Zheng, 2021). Meanwhile, clearer synaptic structures can be observed after GLP-1 RAs treatment (Qi et al., 2016; An et al., 2019; Zheng, 2021). The therapeutic effects of GLP-1 RAs on synapses may involve several aspects. GLP-1 RAs can activate G proteins, which in turn activate the adenylate cyclase system, leading to an increase in cAMP and protein kinase A (PKA). The increase in PKA can lead to an increase in synaptic neurotransmitters and LTP enhancement. Adenosine diphosphate, which is produced by the adenylate cyclase system during cAMP production, acts on ATP-sensitive K^+^ channels. This can promote the opening of Ca^2+^ channels and an increase in Ca^2+^ in the cytosol, which can also promote the release of neurotransmitters (Hölscher and Li, 2010). At the same time, Ca^2+^ acts as a second messenger. When the second messenger signaling pathway is activated, it can promote energy utilization and cell growth, while also reducing cell apoptosis, ultimately restoring synaptic function and improving metabolic capacity (Li et al., 2010; Sharma et al., 2014).

In addition to the mechanisms mentioned above, it is worth noting that several studies have suggested additional potential therapeutic mechanisms of GLP-1 RAs for AD. For example, research indicates that these agents may also address AD by enhancing vascular function (blood flow and blood-brain barrier integrity) and offering protection to nerve cells (Grieco et al., 2019). It is believed that the blood-brain barrier and cerebral blood flow are reduced in AD patients before the appearance of Aβ and p-Tau. Animal studies have demonstrated that liraglutide could restore brain dysfunction and thus treat AD (Kelly et al., 2015; Hachinski et al., 2019; Nation et al., 2019). Furthermore, GLP-1 RAs can promote the proliferation of neural precursor cells and facilitate their differentiation into mature neurons, thereby protecting and increasing the number of neurons in the brain (Grieco et al., 2019). GLP-1 RAs are also believed to increase the levels of Mash1 protein through the activation of the PI3K-Akt pathway, which regulates neurogenesis, leading to improved cognitive function in AD patients.

### 4.2 Clinical research advances

The purpose of animal studies is to contribute to drug development with a view to better application in the clinical setting. Based on research, there are sufficient results to demonstrate that GLP-1 RAs are effective in treating AD animal models. However, it is inconclusive whether the results of animal studies can be validated in the human clinic. We identified five clinical studies of GLP-1 RAs for treating AD on ClinicalTrials.gov (Table 2), a global database of privately and publicly funded clinical studies provided by the US National Library of Medicine. A Phase II clinical study of exenatide on AD patients (NCT01255163) showed that exenatide was safe and well-tolerated in humans. Neuropsychological measures, which assess attention and short-term memory, showed significant improvements in the exenatide group at 6 months compared to the placebo group. Furthermore, although the results showed that exenatide reduced the deposition of Aβ_42_ in plasma neuronal extracellular vesicles, no significant changes were shown for other biomarkers (p-Tau, total tau, etc.). Unfortunately, the project was prematurely discontinued due to the cessation of commercial support by the sponsor, and some patients did not complete the trial. Based on the existing current research results (Mullins et al., 2019), the cerebrospinal fluid level of patients after exenatide administration has improved, but whether it is sufficient to produce a therapeutic effect needs to be verified in larger scale trials. There were two clinical studies on liraglutide for the treatment of AD. A 6-month double-blind clinical trial (NCT01469351) investigated the therapeutic effect of liraglutide on AD. The research (Gejl et al., 2016) showed that although liraglutide treatment prevented the negative changes in brain glucose metabolism and cognition function over time, there were no significant differences in amyloid deposition and cognitive function compared to placebo group. Another study (NCT01843075) randomized 204 patients to receive liraglutide or a placebo. The trial used changes in patients’ brain glucose metabolism rate, cognitive assessment scores, microglial activation levels, and tau and amyloid levels as outcomes of treatment efficacy. The results of this trial have not yet been published. In addition, there are two Phase III clinical trials (NCT04777409 and NCT04777396) on semaglutide for AD are in the recruitment stage. The two studies will test the efficacy of semaglutide in patients with mild cognitive impairment (MCI) or mild AD (with and without significant cerebellar vascular disease). According to published studies, GLP-1 RAs have a good safety profile. In addition, we also noted the limitations of existing experiments with small sample sizes and short experimental periods, which may impact the results. Animal models are essentially reductions of specific pathological manifestations of the disease but it cannot cover the pathophysiology of AD patients, so there is still a certain gap between the results of animal experiments and practical clinical applications (Ferrari et al., 2022). More intensive studies should be conducted in the future to develop clinical translation.

**TABLE 2:** GLP-1 RAs clinical trials on ClinicalTrials.gov.

Study	Drug (dose; duration)	Sample (age, sex)	Outcomes	Status
A Pilot Clinical Trial of Exendin-4 in Alzheimer’s Disease (NCT01255163)	Exenatide (5 mcg or 10 mcg twice daily, s.c.)	*n* = 57 (60 years and older; male and female)	MMSE; ADAS-cog70; CDR Global Score; CDR Sum of Boxes; CSF p181-tau; CSF Aβ42; BMI	Terminated; Has Results
Identifying Potential Effects of Liraglutide on Degenerative Changes (NCT01469351)	Liraglutide (1.8 mg once daily, s.c.)	*n* = 34 (50 years–80 years; male and female)	intra-cerebral amyloid deposit; neuro-psychological tests; the change in glucose uptake in the CNS	Completed
Evaluating Liraglutide in Alzheimer’s Disease (ELAD) (NCT01843075)	Liraglutide (1.8 mg once daily, s.c.)	*n* = 204 (50 years and older; male and female)	Cerebral glucose metabolic rate; z-scores for the ADAS Exec, MRI changes, microglial activation, and CSF markers; the incidence and severity of treatment emergent adverse events; the change in microglial activation, tau deposition, and cortical amyloid	Unknown
A Research Study Investigating Semaglutide in People with Early Alzheimer’s Disease (EVOKE Plus) (NCT04777409)	semaglutide (dose gradually increased to 14 mg once daily; oral)	*n* = 1840 (55 years–85 years; male and female)	CDR-SB score; ADCS-ADLMCI score; time to progression to dementia; ADAS-Cog-13 score; MoCA score; ADCOMS; MMSE; the 10-item NPI score; time to progression in disease stage; Number of TEAEs; high sensitivity C-reactive protein level; time to first occurrence of MACE; time to first occurrence of stroke; EQ-5D-5L proxy score; CDR-SB score; ADCS-ADL-MCI score	Recruiting
A Research Study Investigating Semaglutide in People with Early Alzheimer’s Disease (EVOKE) (NCT04777396)	semaglutide (dose gradually increased to 14 mg once daily; oral)	*n* = 1840 (55 years–85 years; male and female)	CDR-SB score; ADCS-ADLMCI score; time to progression to dementia; ADAS-Cog-13 score; MoCA score; ADCOMS; MMSE; the 10-item NPI score; time to progression in disease stage; Number of TEAEs; high sensitivity C-reactive protein level; time to first occurrence of MACE; time to first occurrence of stroke; EQ-5D-5L proxy score; CDR-SB score; ADCS-ADL-MCI score	Recruiting

Note: MMSE, mini mental state examination; ADAS-cog70, 70-item Alzheimer’s Disease Assessment Scale-cognitive Subscale; CDR, clinical dementia rating; CSF, cerebrospinal fluid; CSF p181-tau, Cerebrospinal Fluid phospho181-tau; CSF Abeta42, Cerebrospinal Fluid Amyloid-beta 42; BMI, body mass index; CDR-SB, Clinical Dementia Rating–Sum of Boxes; ADCS-ADLMCI, Alzheimer’s Disease Cooperative Study Activities of Daily Living Scale for MCI; ADAS-Cog-13, 13-item Alzheimer’s Disease Assessment Scale Cognitive Subscale; MoCA, montreal cognitive assessment; ADCOMS, Alzheimer’s Disease Composite Score; NPI, neuropsychiatric inventory; TEAEs, treatment emergent adverse events; MACE, major adverse cardiovascular event.

It was also observed that the patients recruited for the clinical trials of GLP-1 RAs in the treatment of AD are those with mild AD and MCI. This is consistent with the conclusions of our research, indicating that GLP-1 RAs may have therapeutic effects on early-stage AD. Furthermore, we found that the currently registered clinical trials for exenatide therapy in AD are utilizing doses of 10 μg or 20 μg, which lower than the dosage range calculated in our study. This variance could stem from disparities in animal and human metabolism, pharmacokinetics. Additionally, our study utilized intranasal administration, while clinical application primarily involves subcutaneous injections. These differing administration routes may also contribute to result disparities. Currently, only one clinical study investigating the use of exenatide for AD has been retrieved from ClinicalTrials.gov. Based on the above, the research of exenatide for AD is still in its preliminary stage. Therefore, the findings of this study can provide new insights and references for future research.

Meanwhile, the results of the present study showed that the intranasal administration of exenatide was more effective than subcutaneous administration in the treatment of AD, which provides a new insight for further research. Due to the limited clinical trials, more researches should be carried out, and the introduction of the intranasal administration route should be considered, and the bioavailability and efficacy differences between it and the traditional subcutaneous administration route should be comprehensively compared and explored.

### 4.3 Advantages and limitations

To the best of our knowledge, this is the first meta-analysis of GLP-1 RAs-treated AD animals. This study comprehensively assessed the therapeutic effects of GLP-1 RAs in AD animal models based on two main aspects: behavioral tests and pathological features. We included 26 articles, and overall, the study results provide convincing evidence that GLP-1 RAs are neuroprotective in AD.

However, this study has certain limits, including the fact that the analysis was performed on published literature and may not involve grey literature, thereby leading to biased information. Secondly, the different quality of the included articles, for example, in allocation concealment and blinding, needed to be clearly stated, which to some extent, affected the reliability of the results. There is some heterogeneity among the studies, which may be caused by the type of GLP-1 RAs, the way of administration, the dose of administration, the cycle of administration, the gender of animals, etc. Due to the limited number of included articles, only subgroup analysis was performed based on different modeling methods and animal strains, which may have some influence on the results. We evaluated the funnel plot by the Trim and Fill method, and while the study had some publication bias, the findings were robust and reliable.

## 5 Conclusion

This study comprehensively evaluated the efficacy of GLP-1 RAs in AD animal models. Twenty-six studies were selected from seven databases based on inclusion and exclusion criteria. The meta-analysis found that GLP-1 RAs can significantly improve AD animals’ learning and memory abilities, reducing the deposition of Aβ and hyperphosphorylation of tau protein in the brain. The results indicate that GLP-1 RAs are promising candidate agents for AD treatment, which may improve cognitive performance and alleviate pathological features through multiple mechanisms. Although the study has some limitations, the results of the study are still worthy of attention, and can provide reference for further experimental design and clinical research.

## Data Availability

The original contributions presented in the study are included in the article/Supplementary Material, further inquiries can be directed to the corresponding authors.

## References

[B1] Abbas T. Faivre E. Hölscher C. (2009). Impairment of synaptic plasticity and memory formation in GLP-1 receptor KO mice: Interaction between type 2 diabetes and Alzheimer’s disease. Behav. Brain Res. 205, 265–271. 10.1016/j.bbr.2009.06.035 19573562

[B2] Ahmad W. Ijaz B. Shabbiri K. Ahmed F. Rehman S. (2017). Oxidative toxicity in diabetes and Alzheimer’s disease: Mechanisms behind ROS/RNS generation. J. Biomed. Sci. 24, 76. 10.1186/s12929-017-0379-z 28927401 PMC5606025

[B3] Akiyama H. Barger S. Barnum S. Bradt B. Bauer J. Cole G. M. (2000). Inflammation and Alzheimer’s disease. Neurobiol. Aging 21, 383–421. 10.1016/s0197-4580(00)00124-x 10858586 PMC3887148

[B4] Ali Y. O. Bradley G. Lu H.-C. (2017). Screening with an NMNAT2-MSD platform identifies small molecules that modulate NMNAT2 levels in cortical neurons. Sci. Rep. 7, 43846. 10.1038/srep43846 28266613 PMC5358788

[B5] Alvarez E. Martínez M. D. Roncero I. Chowen J. A. García-Cuartero B. Gispert J. D. (2005). The expression of GLP-1 receptor mRNA and protein allows the effect of GLP-1 on glucose metabolism in the human hypothalamus and brainstem. J. Neurochem. 92, 798–806. 10.1111/j.1471-4159.2004.02914.x 15686481

[B6] Alzheimer’s Association Calcium Hypothesis Workgroup (2017). Calcium Hypothesis of Alzheimer’s disease and brain aging: A framework for integrating new evidence into a comprehensive theory of pathogenesis. Alzheimers Dement. 13, 178–182.e17. 10.1016/j.jalz.2016.12.006 28061328

[B7] An F.-M. Chen S. Xu Z. Yin L. Wang Y. Liu A.-R. (2015). Glucagon-like peptide-1 regulates mitochondrial biogenesis and tau phosphorylation against advanced glycation end product-induced neuronal insult: Studies *in vivo* and *in vitro* . Neuroscience 300, 75–84. 10.1016/j.neuroscience.2015.05.023 25987199

[B8] An J. Zhou Y. Zhang M. Xie Y. Ke S. Liu L. (2019). Exenatide alleviates mitochondrial dysfunction and cognitive impairment in the 5×FAD mouse model of Alzheimer’s disease. Behav. Brain Res. 370, 111932. 10.1016/j.bbr.2019.111932 31082410

[B9] An Y. Varma V. R. Varma S. Casanova R. Dammer E. Pletnikova O. (2018). Evidence for brain glucose dysregulation in Alzheimer’s disease. Alzheimers Dement. 14, 318–329. 10.1016/j.jalz.2017.09.011 29055815 PMC5866736

[B10] Arias C. Becerra-García F. Arrieta I. Tapia R. (1998). The protein phosphatase inhibitor okadaic acid induces heat shock protein expression and neurodegeneration in rat hippocampus *in vivo* . Exp. Neurol. 153, 242–254. 10.1006/exnr.1998.6900 9784284

[B11] Bain S. C. (2014). The clinical development program of lixisenatide: a once-daily glucagon-like peptide-1 receptor agonist. Diabetes Ther. 5, 367–383. 10.1007/s13300-014-0073-z 25027491 PMC4269639

[B12] Bakota L. Brandt R. (2016). Tau biology and tau-directed therapies for Alzheimer’s disease. Drugs 76, 301–313. 10.1007/s40265-015-0529-0 26729186 PMC4757605

[B13] Bassil F. Fernagut P.-O. Bezard E. Meissner W. G. (2014). Insulin, IGF-1 and GLP-1 signaling in neurodegenerative disorders: targets for disease modification? Prog. Neurobiol. 118, 1–18. 10.1016/j.pneurobio.2014.02.005 24582776

[B14] Bateman R. J. Cummings J. Schobel S. Salloway S. Vellas B. Boada M. (2022). Gantenerumab: an anti-amyloid monoclonal antibody with potential disease-modifying effects in early Alzheimer’s disease. Alzheimers Res. Ther. 14, 178. 10.1186/s13195-022-01110-8 36447240 PMC9707418

[B15] Bertilsson G. Patrone C. Zachrisson O. Andersson A. Dannaeus K. Heidrich J. (2008). Peptide hormone exendin-4 stimulates subventricular zone neurogenesis in the adult rodent brain and induces recovery in an animal model of Parkinson’s disease. J. Neurosci. Res. 86, 326–338. 10.1002/jnr.21483 17803225

[B16] Boccardi V. Murasecco I. Mecocci P. (2019). Diabetes drugs in the fight against Alzheimer’s disease. Ageing Res. Rev. 54, 100936. 10.1016/j.arr.2019.100936 31330313

[B17] Bomba M. Granzotto A. Castelli V. Onofrj M. Lattanzio R. Cimini A. (2019). Exenatide reverts the high-fat-diet-induced impairment of BDNF signaling and inflammatory response in an animal model of Alzheimer’s disease. J. Alzheimer’s Dis. 70, 793–810. 10.3233/JAD-190237 31256135

[B18] Bomfim T. R. Forny-Germano L. Sathler L. B. Brito-Moreira J. Houzel J.-C. Decker H. (2012b). An anti-diabetes agent protects the mouse brain from defective insulin signaling caused by Alzheimer’s disease-associated Aβ oligomers. J. Clin. Invest. 122, 1339–1353. 10.1172/JCI57256 22476196 PMC3314445

[B19] Bomfim T. R. Forny-Germano L. Sathler L. B. Brito-Moreira J. Houzel J. C. Decker H. (2012a). An anti-diabetes agent protects the mouse brain from defective insulin signaling caused by Alzheimer’s disease-associated A beta oligomers. J. Clin. Investigation 122, 1339–1353. 10.1172/jci57256 PMC3314445 22476196

[B20] Brodeur J. Thériault C. Lessard-Beaudoin M. Marcil A. Dahan S. Lavoie C. (2012). LDLR-related protein 10 (LRP10) regulates amyloid precursor protein (APP) trafficking and processing: evidence for a role in Alzheimer’s disease. Mol. Neurodegener. 7, 31. 10.1186/1750-1326-7-31 22734645 PMC3520120

[B21] Cai H. Y. Holscher C. Yue X. H. Zhang S. X. Wang X. H. Qiao F. (2014). Lixisenatide rescues spatial memory and synaptic plasticity from amyloid beta protein-induced impairments in rats. Neuroscience 277, 6–13. 10.1016/j.neuroscience.2014.02.022 24583037

[B22] Calsolaro V. Edison P. (2016). Neuroinflammation in Alzheimer’s disease: Current evidence and future directions. Alzheimers Dement. 12, 719–732. 10.1016/j.jalz.2016.02.010 27179961

[B23] Campbell J. E. Drucker D. J. (2013). Pharmacology, physiology, and mechanisms of incretin hormone action. Cell Metab. 17, 819–837. 10.1016/j.cmet.2013.04.008 23684623

[B24] Cantini G. Mannucci E. Luconi M. (2016). Perspectives in GLP-1 research: New targets, new receptors. Trends Endocrinol. Metab. 27, 427–438. 10.1016/j.tem.2016.03.017 27091492

[B25] Chakrabarti S. Khemka V. K. Banerjee A. Chatterjee G. Ganguly A. Biswas A. (2015). Metabolic risk factors of sporadic Alzheimer’s disease: Implications in the pathology, pathogenesis and treatment. Aging Dis. 6, 282–299. 10.14336/AD.2014.002 26236550 PMC4509477

[B26] Chen K. Zhao X.-L. Li L.-B. Huang L.-Y. Tang Z. Luo J. (2020). miR-503/Apelin-12 mediates high glucose-induced microvascular endothelial cells injury via JNK and p38MAPK signaling pathway. Regen. Ther. 14, 111–118. 10.1016/j.reth.2019.12.002 31989001 PMC6970136

[B27] Chen S. Y. Sun J. Zhao G. Guo A. Chen Y. L. Fu R. X. (2017). Liraglutide improves water maze learning and memory performance while reduces hyperphosphorylation of tau and neurofilaments in APP/PS1/tau triple transgenic mice. Neurochem. Res. 42, 2326–2335. 10.1007/s11064-017-2250-8 28382596

[B28] Chen Y. Liang Z. Blanchard J. Dai C.-L. Sun S. Lee M. H. (2013). A non-transgenic mouse model (icv-STZ mouse) of Alzheimer’s disease: similarities to and differences from the transgenic model (3xTg-AD mouse). Mol. Neurobiol. 47, 711–725. 10.1007/s12035-012-8375-5 23150171 PMC3582864

[B29] Cheng D. Yang S. Zhao X. Wang G. (2022). The role of glucagon-like peptide-1 receptor agonists (GLP-1 RA) in diabetes-related neurodegenerative diseases. Drug Des. Devel Ther. 16, 665–684. 10.2147/DDDT.S348055 PMC8943601 35340338

[B30] Chiang H.-C. Wang L. Xie Z. Yau A. Zhong Y. (2010). PI3 kinase signaling is involved in Abeta-induced memory loss in Drosophila. Proc. Natl. Acad. Sci. U. S. A. 107, 7060–7065. 10.1073/pnas.0909314107 20351282 PMC2872421

[B31] Chiu S.-L. Chen C.-M. Cline H. T. (2008). Insulin receptor signaling regulates synapse number, dendritic plasticity, and circuit function *in vivo* . Neuron 58, 708–719. 10.1016/j.neuron.2008.04.014 18549783 PMC3057650

[B32] Corder E. H. Ghebremedhin E. Taylor M. G. Thal D. R. Ohm T. G. Braak H. (2004). The biphasic relationship between regional brain senile plaque and neurofibrillary tangle distributions: Modification by age, sex, and APOE polymorphism. Ann. N. Y. Acad. Sci. 1019, 24–28. 10.1196/annals.1297.005 15246987

[B33] Correia S. C. Santos R. X. Santos M. S. Casadesus G. Lamanna J. C. Perry G. (2013). Mitochondrial abnormalities in a streptozotocin-induced rat model of sporadic Alzheimer’s disease. Curr. Alzheimer Res. 10, 406–419. 10.2174/1567205011310040006 23061885

[B34] Deacon C. F. Johnsen A. H. Holst J. J. (1995). Degradation of glucagon-like peptide-1 by human plasma *in vitro* yields an N-terminally truncated peptide that is a major endogenous metabolite *in vivo* . J. Clin. Endocrinol. Metab. 80, 952–957. 10.1210/jcem.80.3.7883856 7883856

[B35] Diz-Chaves Y. Herrera-Pérez S. González-Matías L. C. Mallo F. (2022a). Effects of Glucagon-like peptide 1 (GLP-1) analogs in the hippocampus. Vitam. Horm. 118, 457–478. 10.1016/bs.vh.2021.12.005 35180937

[B36] Diz-Chaves Y. Mastoor Z. Spuch C. González-Matías L. C. Mallo F. (2022b). Anti-inflammatory effects of GLP-1 receptor activation in the brain in neurodegenerative diseases. Int. J. Mol. Sci. 23, 9583. 10.3390/ijms23179583 36076972 PMC9455625

[B37] Dou J.-T. Chen M. Dufour F. Alkon D. L. Zhao W.-Q. (2005). Insulin receptor signaling in long-term memory consolidation following spatial learning. Learn Mem. 12, 646–655. 10.1101/lm.88005 16287721 PMC1356184

[B38] Drummond E. Wisniewski T. (2017). Alzheimer’s disease: experimental models and reality. Acta Neuropathol. 133, 155–175. 10.1007/s00401-016-1662-x 28025715 PMC5253109

[B39] Du H. Guo L. Yan S. Sosunov A. A. McKhann G. M. Yan S. S. (2010). Early deficits in synaptic mitochondria in an Alzheimer’s disease mouse model. Proc. Natl. Acad. Sci. U. S. A. 107, 18670–18675. 10.1073/pnas.1006586107 20937894 PMC2972922

[B40] Duelli R. Schröck H. Kuschinsky W. Hoyer S. (1994). Intracerebroventricular injection of streptozotocin induces discrete local changes in cerebral glucose utilization in rats. Int. J. Dev. Neurosci. 12, 737–743. 10.1016/0736-5748(94)90053-1 7747600

[B41] During M. J. Cao L. Zuzga D. S. Francis J. S. Fitzsimons H. L. Jiao X. (2003). Glucagon-like peptide-1 receptor is involved in learning and neuroprotection. Nat. Med. 9, 1173–1179. 10.1038/nm919 12925848

[B42] Egefjord L. Gejl M. Møller A. Brændgaard H. Gottrup H. Antropova O. (2012). Effects of liraglutide on neurodegeneration, blood flow and cognition in Alzheimer´s disease - protocol for a controlled, randomized double-blinded trial. Dan. Med. J. 59, A4519. 23158895

[B43] Esquerda-Canals G. Montoliu-Gaya L. Güell-Bosch J. Villegas S. (2017). Mouse models of Alzheimer’s disease. J. Alzheimers Dis. 57, 1171–1183. 10.3233/JAD-170045 28304309

[B44] Ferrari F. Moretti A. Villa R. F. (2022). Incretin-based drugs as potential therapy for neurodegenerative diseases: current status and perspectives. Pharmacol. Ther. 239, 108277. 10.1016/j.pharmthera.2022.108277 36064147

[B45] Fillit H. Ding W. H. Buee L. Kalman J. Altstiel L. Lawlor B. (1991). Elevated circulating tumor necrosis factor levels in Alzheimer’s disease. Neurosci. Lett. 129, 318–320. 10.1016/0304-3940(91)90490-k 1745413

[B46] Finan B. Clemmensen C. Müller T. D. (2015). Emerging opportunities for the treatment of metabolic diseases: Glucagon-like peptide-1 based multi-agonists. Mol. Cell Endocrinol. 418 (1), 42–54. 10.1016/j.mce.2015.07.003 26151488

[B47] Forner S. Baglietto-Vargas D. Martini A. C. Trujillo-Estrada L. LaFerla F. M. (2017). Synaptic impairment in Alzheimer’s disease: A dysregulated symphony. Trends Neurosci. 40, 347–357. 10.1016/j.tins.2017.04.002 28494972

[B48] Fu W.-Y. Ip N. Y. (2022). The role of genetic risk factors of Alzheimer’s disease in synaptic dysfunction. Semin. Cell Dev. Biol. S1084-9521 (22), 3–12. 10.1016/j.semcdb.2022.07.011 35918217

[B49] Gao C. Liu Y. Jiang Y. Ding J. Li L. (2014). Geniposide ameliorates learning memory deficits, reduces tau phosphorylation and decreases apoptosis via GSK3β pathway in streptozotocin-induced alzheimer rat model. Brain Pathol. 24, 261–269. 10.1111/bpa.12116 24329968 PMC8029432

[B50] Garabadu D. Verma J. (2019). Exendin-4 attenuates brain mitochondrial toxicity through PI3K/Akt-dependent pathway in amyloid beta (1-42)-induced cognitive deficit rats. Neurochem. Int. 128, 39–49. 10.1016/j.neuint.2019.04.006 31004737

[B51] Gejl M. Gjedde A. Egefjord L. Møller A. Hansen S. B. Vang K. (2016). In Alzheimer’s disease, 6-month treatment with GLP-1 analog prevents decline of brain glucose metabolism: Randomized, placebo-controlled, double-blind clinical trial. Front. Aging Neurosci. 8, 108. 10.3389/fnagi.2016.00108 27252647 PMC4877513

[B52] Götz J. Ittner L. M. (2008). Animal models of Alzheimer’s disease and frontotemporal dementia. Nat. Rev. Neurosci. 9, 532–544. 10.1038/nrn2420 18568014

[B53] Gourmaud S. Paquet C. Dumurgier J. Pace C. Bouras C. Gray F. (2015). Increased levels of cerebrospinal fluid JNK3 associated with amyloid pathology: Links to cognitive decline. J. Psychiatry Neurosci. 40, 151–161. 10.1503/jpn.140062 25455349 PMC4409432

[B54] Green B. D. Lavery K. S. Irwin N. O’harte F. P. M. Harriott P. Greer B. (2006). Novel glucagon-like peptide-1 (GLP-1) analog (Val8)GLP-1 results in significant improvements of glucose tolerance and pancreatic beta-cell function after 3-week daily administration in obese diabetic (ob/ob) mice. J. Pharmacol. Exp. Ther. 318, 914–921. 10.1124/jpet.105.097824 16648370

[B55] Grieb P. Kryczka T. Fiedorowicz M. Frontczak-Baniewicz M. Walski M. (2004). Expansion of the Golgi apparatus in rat cerebral cortex following intracerebroventricular injections of streptozotocin. Acta Neurobiol. Exp. (Wars) 64, 481–489. 15586665 10.55782/ane-2004-1531

[B56] Grieco M. Giorgi A. Gentile M. C. d’Erme M. Morano S. Maras B. (2019). Glucagon-like peptide-1: A focus on neurodegenerative diseases. Front. Neurosci. 13, 1112. 10.3389/fnins.2019.01112 31680842 PMC6813233

[B57] Griffith C. M. Eid T. Rose G. M. Patrylo P. R. (2018). Evidence for altered insulin receptor signaling in Alzheimer’s disease. Neuropharmacology 136, 202–215. 10.1016/j.neuropharm.2018.01.008 29353052

[B58] Guo J.-T. Yu J. Grass D. de Beer F. C. Kindy M. S. (2002). Inflammation-dependent cerebral deposition of serum amyloid a protein in a mouse model of amyloidosis. J. Neurosci. 22, 5900–5909. 12122052 10.1523/JNEUROSCI.22-14-05900.2002 PMC6757908

[B59] Guo L. Cao J. Hou J. Li Y. Huang M. Zhu L. (2023). Sex specific molecular networks and key drivers of Alzheimer’s disease. Mol. Neurodegener. 18, 39. 10.1186/s13024-023-00624-5 37340466 PMC10280841

[B60] Guo L. Zhong M. B. Zhang L. Zhang B. Cai D. (2022). Sex differences in Alzheimer’s disease: Insights from the multiomics landscape. Biol. Psychiatry 91, 61–71. 10.1016/j.biopsych.2021.02.968 33896621 PMC8996342

[B61] Hachinski V. Einhäupl K. Ganten D. Alladi S. Brayne C. Stephan B. C. M. (2019). Preventing dementia by preventing stroke: The Berlin Manifesto. Alzheimers Dement. 15, 961–984. 10.1016/j.jalz.2019.06.001 31327392 PMC7001744

[B62] Hamilto A. Holscher C. (2009). Receptors for the incretin glucagon-like peptide-1 are expressed on neurons in the central nervous system. Neuroreport 20, 1161–1166. 10.1097/WNR.0b013e32832fbf14 19617854

[B63] Hamilton A. Patterson S. Porter D. Gault V. A. Holscher C. (2011). Novel GLP-1 mimetics developed to treat type 2 diabetes promote progenitor cell proliferation in the brain. J. Neurosci. Res. 89, 481–489. 10.1002/jnr.22565 21312223

[B64] Han W. N. Hölscher C. Yuan L. Yang W. Wang X. H. Wu M. N. (2013). Liraglutide protects against amyloid-β protein-induced impairment of spatial learning and memory in rats. Neurobiol. Aging 34, 576–588. 10.1016/j.neurobiolaging.2012.04.009 22592020

[B65] Hansen H. H. Fabricius K. Barkholt P. Kongsbak-Wismann P. Schlumberger C. Jelsing J. (2016). Long-term treatment with liraglutide, a glucagon-like peptide-1 (GLP-1) receptor agonist, has no effect on β-amyloid plaque load in two transgenic APP/PS1 mouse models of Alzheimer’s disease. PLoS ONE 11, e0158205. 10.1371/journal.pone.0158205 27421117 PMC4946784

[B66] Harr S. D. Simonian N. A. Hyman B. T. (1995). Functional alterations in Alzheimer’s disease: decreased glucose transporter 3 immunoreactivity in the perforant pathway terminal zone. J. Neuropathol. Exp. Neurol. 54, 38–41. 10.1097/00005072-199501000-00005 7815078

[B67] Harris P. L. Zhu X. Pamies C. Rottkamp C. A. Ghanbari H. A. McShea A. (2000). Neuronal polo-like kinase in Alzheimer disease indicates cell cycle changes. Neurobiol. Aging 21, 837–841. 10.1016/s0197-4580(00)00218-9 11124427

[B69] Hölscher C. (2020). Brain insulin resistance: role in neurodegenerative disease and potential for targeting. Expert Opin. Investig. Drugs 29, 333–348. 10.1080/13543784.2020.1738383 32175781

[B70] Hölscher C. Li L. (2010). New roles for insulin-like hormones in neuronal signalling and protection: new hopes for novel treatments of Alzheimer’s disease? Neurobiol. Aging 31, 1495–1502. 10.1016/j.neurobiolaging.2008.08.023 18930564

[B71] Hooijmans C. R. Rovers M. M. de Vries R. B. M. Leenaars M. Ritskes-Hoitinga M. Langendam M. W. (2014). SYRCLE’s risk of bias tool for animal studies. BMC Med. Res. Methodol. 14, 43. 10.1186/1471-2288-14-43 24667063 PMC4230647

[B72] Hou Y. C. Tsai S. Y. Lai P. Y. Chen Y. S. Chao P. D. L. (2008). Metabolism and pharmacokinetics of genipin and geniposide in rats. Food Chem. Toxicol. 46, 2764–2769. 10.1016/j.fct.2008.04.033 18550245

[B73] Hoyer S. Müller D. Plaschke K. (1994). Desensitization of brain insulin receptor. Effect on glucose/energy and related metabolism. J. Neural Transm. Suppl. 44, 259–268. 10.1007/978-3-7091-9350-1_20 7897397

[B74] Hoyer S. Oesterreich K. Wagner O. (1988). Glucose metabolism as the site of the primary abnormality in early-onset dementia of Alzheimer type? J. Neurol. 235, 143–148. 10.1007/BF00314304 3367161

[B75] Hoyer S. Prem L. Sorbi S. Amaducci L. (1993). Stimulation of glycolytic key enzymes in cerebral cortex by insulin. Neuroreport 4, 991–993. 10.1097/00001756-199307000-00039 8369496

[B76] Hubbard S. R. (1997). Crystal structure of the activated insulin receptor tyrosine kinase in complex with peptide substrate and ATP analog. EMBO J. 16, 5572–5581. 10.1093/emboj/16.18.5572 9312016 PMC1170189

[B77] Hubbard S. R. (2013). The insulin receptor: both a prototypical and atypical receptor tyrosine kinase. Cold Spring Harb. Perspect. Biol. 5, a008946. 10.1101/cshperspect.a008946 23457259 PMC3578362

[B78] Iannuzzi C. Irace G. Sirangelo I. (2014). Differential effects of glycation on protein aggregation and amyloid formation. Front. Mol. Biosci. 1, 9. 10.3389/fmolb.2014.00009 25988150 PMC4428487

[B79] Irizarry M. C. McNamara M. Fedorchak K. Hsiao K. Hyman B. T. (1997). APPSw transgenic mice develop age-related A beta deposits and neuropil abnormalities, but no neuronal loss in CA1. J. Neuropathol. Exp. Neurol. 56, 965–973. 10.1097/00005072-199709000-00002 9291938

[B80] Janhavi P. Divyashree S. Sanjailal K. P. Muthukumar S. P. (2022). DoseCal: a virtual calculator for dosage conversion between human and different animal species. Arch. Physiol. Biochem. 128, 426–430. 10.1080/13813455.2019.1687523 31746232

[B81] Jantrapirom S. Nimlamool W. Chattipakorn N. Chattipakorn S. Temviriyanukul P. Inthachat W. (2020). Liraglutide suppresses tau hyperphosphorylation, amyloid beta accumulation through regulating neuronal insulin signaling and BACE-1 activity. Int. J. Mol. Sci. 21, 1725. 10.3390/ijms21051725 32138327 PMC7084306

[B82] Jeremic D. Jiménez-Díaz L. Navarro-López J. D. (2021). Past, present and future of therapeutic strategies against amyloid-β peptides in Alzheimer’s disease: a systematic review. Ageing Res. Rev. 72, 101496. 10.1016/j.arr.2021.101496 34687956

[B83] Jha M. K. Jo M. Kim J.-H. Suk K. (2019). Microglia-astrocyte crosstalk: An intimate molecular conversation. Neuroscientist 25, 227–240. 10.1177/1073858418783959 29931997

[B84] Jia X. T. Ye T. Yuan L. Zhang G. J. Liu Z. Q. Di Z. L. (2016). Exendin-4, a glucagon-like peptide 1 receptor agonist, protects against amyloid-beta peptide-induced impairment of spatial learning and memory in rats. Physiology Behav. 159, 72–79. 10.1016/j.physbeh.2016.03.016 26992957

[B85] Jia-Yue C. Zhu Q. Zhang S. Ouyang D. Lu J-H. (2019). Resveratrol in experimental Alzheimer’s disease models: A systematic review of preclinical studies. Pharmacol. Res. 150, 104476. 10.1016/j.phrs.2019.104476 31605783

[B86] John A. Reddy P. H. (2021). Synaptic basis of Alzheimer’s disease: Focus on synaptic amyloid beta, P-tau and mitochondria. Ageing Res. Rev. 65, 101208. 10.1016/j.arr.2020.101208 33157321 PMC7770124

[B68] Higgins J. Green S. (Editors) (2011). Cochrane handbook for systematic reviews of interventions. Version 5.1.0.

[B87] Kamat P. K. Rai S. Nath C. (2013). Okadaic acid induced neurotoxicity: an emerging tool to study Alzheimer’s disease pathology. Neurotoxicology 37, 163–172. 10.1016/j.neuro.2013.05.002 23688530

[B88] Kamat P. K. Rai S. Swarnakar S. Shukla R. Nath C. (2014). Molecular and cellular mechanism of okadaic acid (OKA)-induced neurotoxicity: a novel tool for Alzheimer’s disease therapeutic application. Mol. Neurobiol. 50, 852–865. 10.1007/s12035-014-8699-4 24710687

[B89] Kannanayakal T. J. Tao H. Vandre D. D. Kuret J. (2006). Casein kinase-1 isoforms differentially associate with neurofibrillary and granulovacuolar degeneration lesions. Acta Neuropathol. 111, 413–421. 10.1007/s00401-006-0049-9 16557393

[B90] Kawasaki N. Asada R. Saito A. Kanemoto S. Imaizumi K. (2012). Obesity-induced endoplasmic reticulum stress causes chronic inflammation in adipose tissue. Sci. Rep. 2, 799. 10.1038/srep00799 23150771 PMC3495279

[B91] Kelly P. McClean P. L. Ackermann M. Konerding M. A. Hölscher C. Mitchell C. A. (2015). Restoration of cerebral and systemic microvascular architecture in APP/PS1 transgenic mice following treatment with Liraglutide^TM^ . Microcirculation 22, 133–145. 10.1111/micc.12186 25556713

[B92] Khan S. Barve K. H. Kumar M. S. (2020). Recent advancements in pathogenesis, diagnostics and treatment of Alzheimer’s disease. Curr. Neuropharmacol. 18, 1106–1125. 10.2174/1570159X18666200528142429 32484110 PMC7709159

[B93] Kieffer T. J. Habener J. F. (1999). The glucagon-like peptides. Endocr. Rev. 20, 876–913. 10.1210/edrv.20.6.0385 10605628

[B94] Kleinridders A. Ferris H. A. Cai W. Kahn C. R. (2014). Insulin action in brain regulates systemic metabolism and brain function. Diabetes 63, 2232–2243. 10.2337/db14-0568 24931034 PMC4066341

[B95] Klosinski L. P. Yao J. Yin F. Fonteh A. N. Harrington M. G. Christensen T. A. (2015). White matter lipids as a ketogenic fuel supply in aging female brain: Implications for Alzheimer’s disease. EBioMedicine 2, 1888–1904. 10.1016/j.ebiom.2015.11.002 26844268 PMC4703712

[B96] Knopman D. S. Amieva H. Petersen R. C. Chételat G. Holtzman D. M. Hyman B. T. (2021). Alzheimer disease. Nat. Rev. Dis. Prim. 7, 33. 10.1038/s41572-021-00269-y 33986301 PMC8574196

[B97] Kurochkin I. V. Guarnera E. Berezovsky I. N. (2018). Insulin-degrading enzyme in the fight against Alzheimer’s disease. Trends Pharmacol. Sci. 39, 49–58. 10.1016/j.tips.2017.10.008 29132916

[B98] Lee V. M. Goedert M. Trojanowski J. Q. (2001). Neurodegenerative tauopathies. Annu. Rev. Neurosci. 24, 1121–1159. 10.1146/annurev.neuro.24.1.1121 11520930

[B99] Lennox R. Porter D. W. Flatt P. R. Gault V. A. (2013). Val(8))GLP-1-Glu-PAL: a GLP-1 agonist that improves hippocampal neurogenesis, glucose homeostasis, and β-cell function in high-fat-fed mice. ChemMedChem 8, 595–602. 10.1002/cmdc.201200409 23138973

[B100] Lester-Coll N. Rivera E. J. Soscia S. J. Doiron K. Wands J. R. de la Monte S. M. (2006). Intracerebral streptozotocin model of type 3 diabetes: relevance to sporadic Alzheimer’s disease. J. Alzheimers Dis. 9, 13–33. 10.3233/jad-2006-9102 16627931

[B101] Li J. Li N. Yan S. Lu Y. Miao X. Gu Z. (2019a). Liraglutide protects renal mesangial cells against hyperglycemia-mediated mitochondrial apoptosis by activating the ERK-Yap signaling pathway and upregulating Sirt3 expression. Mol. Med. Rep. 19, 2849–2860. 10.3892/mmr.2019.9946 30816450

[B102] Li J. X. Wei Y. F. Yin P. Liu L. Lu C. Gong Z. Z. (2019b). Exploration of neuroprotective mechanism of GLP-1 in animal models with Alzheimer’s disease. Int. J. Clin. Exp. Med. 12, 3897–3904.

[B103] Li Y. Tweedie D. Mattson M. P. Holloway H. W. Greig N. H. (2010). Enhancing the GLP-1 receptor signaling pathway leads to proliferation and neuroprotection in human neuroblastoma cells. J. Neurochem. 113, 1621–1631. 10.1111/j.1471-4159.2010.06731.x 20374430 PMC2912144

[B104] Liu J. Zheng X. Yin F. Hu Y. Guo L. Deng X. (2006). Neurotrophic property of geniposide for inducing the neuronal differentiation of PC12 cells. Int. J. Dev. Neurosci. 24, 419–424. 10.1016/j.ijdevneu.2006.08.009 17045447

[B105] Lu C. Li X. Sun Y. Liu L. Wei Y. (2019). The effect and mechanism of Glucagon like peptide 1(GLP-1)in improving cognitive function of Alzheimer’s disease rat model. Stroke Nerv. Dis. 26, 193–197. 10.3969/j.issn.1007-0478.2019.02.015

[B106] Lynn J. Park M. Ogunwale C. Acquaah-Mensah G. K. (2022). A tale of two diseases: Exploring mechanisms linking diabetes mellitus with Alzheimer’s disease. J. Alzheimers Dis. 85, 485–501. 10.3233/JAD-210612 34842187

[B107] Mauvais-Jarvis F. Clegg D. J. Hevener A. L. (2013). The role of estrogens in control of energy balance and glucose homeostasis. Endocr. Rev. 34, 309–338. 10.1210/er.2012-1055 23460719 PMC3660717

[B108] Malm-Erjefält M. Bjørnsdottir I. Vanggaard J. Helleberg H. Larsen U. Oosterhuis B. (2010). Metabolism and excretion of the once-daily human glucagon-like peptide-1 analog liraglutide in healthy male subjects and its *in vitro* degradation by dipeptidyl peptidase IV and neutral endopeptidase. Drug Metab. Dispos. 38, 1944–1953. 10.1124/dmd.110.034066 20709939

[B109] Mangialasche F. Solomon A. Winblad B. Mecocci P. Kivipelto M. (2010). Alzheimer’s disease: clinical trials and drug development. Lancet Neurol. 9, 702–716. 10.1016/S1474-4422(10)70119-8 20610346

[B110] Matsuoka Y. Yamashita A. Matsuda M. Kawai K. Sawa T. Amaya F. (2019). NLRP2 inflammasome in dorsal root ganglion as a novel molecular platform that produces inflammatory pain hypersensitivity. Pain 160, 2149–2160. 10.1097/j.pain.0000000000001611 31162334

[B111] McClean P. L. Hölscher C. (2014). Liraglutide can reverse memory impairment, synaptic loss and reduce plaque load in aged APP/PS1 mice, a model of Alzheimer’s disease. Neuropharmacology 76, 57–67. 10.1016/j.neuropharm.2013.08.005 23973293

[B112] McClean P. L. Jalewa J. Holscher C. (2015). Prophylactic liraglutide treatment prevents amyloid plaque deposition, chronic inflammation and memory impairment in APP/PS1 mice. Behav. Brain Res. 293, 96–106. 10.1016/j.bbr.2015.07.024 26205827

[B113] McClean P. L. Parthsarathy V. Faivre E. Hölscher C. (2011). The diabetes drug liraglutide prevents degenerative processes in a mouse model of Alzheimer’s disease. J. Neurosci. 31, 6587–6594. 10.1523/jneurosci.0529-11.2011 21525299 PMC6622662

[B114] Mistur R. Mosconi L. Santi S. D. Guzman M. Li Y. Tsui W. (2009). Current challenges for the early detection of Alzheimer’s disease: Brain imaging and CSF studies. J. Clin. Neurol. 5, 153–166. 10.3988/jcn.2009.5.4.153 20076796 PMC2806537

[B115] Mosconi L. Pupi A. De Leon M. J. (2008). Brain glucose hypometabolism and oxidative stress in preclinical Alzheimer’s disease. Ann. N. Y. Acad. Sci. 1147, 180–195. 10.1196/annals.1427.007 19076441 PMC2661241

[B116] Müller T. D. Finan B. Bloom S. R. D’Alessio D. Drucker D. J. Flatt P. R. (2019). Glucagon-like peptide 1 (GLP-1). Mol. Metab. 30, 72–130. 10.1016/j.molmet.2019.09.010 31767182 PMC6812410

[B117] Mullins R. J. Mustapic M. Chia C. W. Carlson O. Gulyani S. Tran J. (2019). A pilot study of exenatide actions in Alzheimer’s disease. Curr. Alzheimer Res. 16, 741–752. 10.2174/1567205016666190913155950 31518224 PMC7476877

[B118] Nakatani Y. Kaneto H. Kawamori D. Yoshiuchi K. Hatazaki M. Matsuoka T. (2005). Involvement of endoplasmic reticulum stress in insulin resistance and diabetes. J. Biol. Chem. 280, 847–851. 10.1074/jbc.M411860200 15509553

[B119] Nation D. A. Sweeney M. D. Montagne A. Sagare A. P. D’Orazio L. M. Pachicano M. (2019). Blood-brain barrier breakdown is an early biomarker of human cognitive dysfunction. Nat. Med. 25, 270–276. 10.1038/s41591-018-0297-y 30643288 PMC6367058

[B120] Nguyen T. T. Ta Q. T. H. Nguyen T. K. O. Nguyen T. T. D. Giau V. V. (2020). Type 3 diabetes and its role implications in Alzheimer’s disease. Int. J. Mol. Sci. 21, 3165. 10.3390/ijms21093165 32365816 PMC7246646

[B121] Nitsch R. Hoyer S. (1991). Local action of the diabetogenic drug, streptozotocin, on glucose and energy metabolism in rat brain cortex. Neurosci. Lett. 128, 199–202. 10.1016/0304-3940(91)90260-z 1834965

[B122] Noble W. Hanger D. P. Miller C. C. J. Lovestone S. (2013). The importance of tau phosphorylation for neurodegenerative diseases. Front. Neurol. 4, 83. 10.3389/fneur.2013.00083 23847585 PMC3696910

[B123] Oddo S. Caccamo A. Shepherd J. D. Murphy M. P. Golde T. E. Kayed R. (2003). Triple-transgenic model of Alzheimer’s disease with plaques and tangles: intracellular Abeta and synaptic dysfunction. Neuron 39, 409–421. 10.1016/s0896-6273(03)00434-3 12895417

[B124] Pantoni M. M. Anagnostaras S. G. (2019). Cognitive effects of mdma in laboratory animals: A systematic review focusing on dose. Pharmacol. Rev. 71, 413–449. 10.1124/pr.118.017087 31249067 PMC6607799

[B125] Patrone C. Eriksson O. Lindholm D. (2014). Diabetes drugs and neurological disorders: new views and therapeutic possibilities. Lancet Diabetes Endocrinol. 2, 256–262. 10.1016/S2213-8587(13)70125-6 24622756

[B126] Pelle M. C. Zaffina I. Giofrè F. Pujia R. Arturi F. (2023). Potential role of glucagon-like peptide-1 receptor agonists in the treatment of cognitive decline and dementia in diabetes mellitus. Int. J. Mol. Sci. 24, 11301. 10.3390/ijms241411301 37511061 PMC10379573

[B127] Prince M. J. Wimo A. Guerchet M. M. Ali G. C. Wu Y.-T. Prina M. (2015). World alzheimer report 2015 - the global impact of dementia: An analysis of prevalence, incidence, cost and trends. Available at: https://kclpure.kcl.ac.uk/portal/en/publications/world-alzheimer-report-2015--the-global-impact-of-dementia(ae525fda-1938-4892-8daa-a2222a672254).html (Accessed November 14, 2022).

[B128] Puzzo D. Gulisano W. Palmeri A. Arancio O. (2015). Rodent models for Alzheimer’s disease drug discovery. Expert Opin. Drug Discov. 10, 703–711. 10.1517/17460441.2015.1041913 25927677 PMC4592281

[B129] Qi L. Ke L. Liu X. Liao L. Ke S. Liu X. (2016). Subcutaneous administration of liraglutide ameliorates learning and memory impairment by modulating tau hyperphosphorylation via the glycogen synthase kinase-3β pathway in an amyloid β protein induced alzheimer disease mouse model. Eur. J. Pharmacol. 783, 23–32. 10.1016/j.ejphar.2016.04.052 27131827

[B130] Querfurth H. W. LaFerla F. M. (2010). Alzheimer’s disease. N. Engl. J. Med. 362, 329–344. 10.1056/NEJMra0909142 20107219

[B131] Radde R. Bolmont T. Kaeser S. A. Coomaraswamy J. Lindau D. Stoltze L. (2006). Abeta42-driven cerebral amyloidosis in transgenic mice reveals early and robust pathology. EMBO Rep. 7, 940–946. 10.1038/sj.embor.7400784 16906128 PMC1559665

[B132] Reddy P. H. Reddy T. P. (2011). Mitochondria as a therapeutic target for aging and neurodegenerative diseases. Curr. Alzheimer Res. 8, 393–409. 10.2174/156720511795745401 21470101 PMC3295247

[B133] Reddy V. P. Zhu X. Perry G. Smith M. A. (2009). Oxidative stress in diabetes and Alzheimer’s disease. J. Alzheimers Dis. 16, 763–774. 10.3233/JAD-2009-1013 19387111 PMC2765716

[B134] Reiman E. M. Chen K. Alexander G. E. Caselli R. J. Bandy D. Osborne D. (2004). Functional brain abnormalities in young adults at genetic risk for late-onset Alzheimer’s dementia. Proc. Natl. Acad. Sci. U. S. A. 101, 284–289. 10.1073/pnas.2635903100 14688411 PMC314177

[B135] Richard B. C. Kurdakova A. Baches S. Bayer T. A. Weggen S. Wirths O. (2015). Gene dosage dependent aggravation of the neurological phenotype in the 5XFAD mouse model of Alzheimer’s disease. J. Alzheimers Dis. 45, 1223–1236. 10.3233/JAD-143120 25697701

[B136] Richard J. E. Anderberg R. H. López-Ferreras L. Olandersson K. Skibicka K. P. (2016). Sex and estrogens alter the action of glucagon-like peptide-1 on reward. Biol. Sex. Differ. 7, 6. 10.1186/s13293-016-0059-9 26779332 PMC4715328

[B137] Rivera E. J. Goldin A. Fulmer N. Tavares R. Wands J. R. de la Monte S. M. (2005). Insulin and insulin-like growth factor expression and function deteriorate with progression of Alzheimer’s disease: Link to brain reductions in acetylcholine. J. Alzheimers Dis. 8, 247–268. 10.3233/jad-2005-8304 16340083

[B138] Robinson A. Lubitz I. Atrakchi-Baranes D. Licht-Murava A. Katsel P. Leroith D. (2019). Combination of insulin with a GLP1 agonist is associated with better memory and normal expression of insulin receptor pathway genes in a mouse model of Alzheimer’s disease. J. Mol. Neurosci. 67, 504–510. 10.1007/s12031-019-1257-9 30635783 PMC6549496

[B139] Root-Bernstein R. Podufaly A. Dillon P. F. (2014). Estradiol binds to insulin and insulin receptor decreasing insulin binding *in vitro* . Front. Endocrinol. (Lausanne) 5, 118. 10.3389/fendo.2014.00118 25101056 PMC4104309

[B140] Salcedo I. Tweedie D. Li Y. Greig N. H. (2012). Neuroprotective and neurotrophic actions of glucagon-like peptide-1: an emerging opportunity to treat neurodegenerative and cerebrovascular disorders. Br. J. Pharmacol. 166, 1586–1599. 10.1111/j.1476-5381.2012.01971.x 22519295 PMC3419902

[B141] Salkovic-Petrisic M. Knezovic A. Hoyer S. Riederer P. (2013). What have we learned from the streptozotocin-induced animal model of sporadic Alzheimer’s disease, about the therapeutic strategies in Alzheimer’s research. J. Neural Transm. (Vienna) 120, 233–252. 10.1007/s00702-012-0877-9 22886150

[B142] Salkovic-Petrisic M. Osmanovic J. Grünblatt E. Riederer P. Hoyer S. (2009). Modeling sporadic Alzheimer’s disease: the insulin resistant brain state generates multiple long-term morphobiological abnormalities including hyperphosphorylated tau protein and amyloid-beta. J. Alzheimers Dis. 18, 729–750. 10.3233/JAD-2009-1184 19661616

[B143] Sastre M. Klockgether T. Heneka M. T. (2006). Contribution of inflammatory processes to Alzheimer’s disease: Molecular mechanisms. Int. J. Dev. Neurosci. 24, 167–176. 10.1016/j.ijdevneu.2005.11.014 16472958

[B144] Sato H. Taketomi Y. Murakami M. (2016). Metabolic regulation by secreted phospholipase A2. Inflamm. Regen. 36, 7. 10.1186/s41232-016-0012-7 29259680 PMC5725825

[B145] Saxena G. Patro I. K. Nath C. (2011). ICV STZ induced impairment in memory and neuronal mitochondrial function: A protective role of nicotinic receptor. Behav. Brain Res. 224, 50–57. 10.1016/j.bbr.2011.04.039 21620901

[B146] Sayre L. M. Lin D. Yuan Q. Zhu X. Tang X. (2006). Protein adducts generated from products of lipid oxidation: focus on HNE and one. Drug Metab. Rev. 38, 651–675. 10.1080/03602530600959508 17145694

[B147] Scheff S. W. Price D. A. Schmitt F. A. Mufson E. J. (2006). Hippocampal synaptic loss in early Alzheimer’s disease and mild cognitive impairment. Neurobiol. Aging 27, 1372–1384. 10.1016/j.neurobiolaging.2005.09.012 16289476

[B148] Scheltens P. De Strooper B. Kivipelto M. Holstege H. Chételat G. Teunissen C. E. (2021). Alzheimer’s disease. Lancet 397, 1577–1590. 10.1016/S0140-6736(20)32205-4 33667416 PMC8354300

[B149] Schönfeld P. Reiser G. (2013). Why does brain metabolism not favor burning of fatty acids to provide energy? Reflections on disadvantages of the use of free fatty acids as fuel for brain. J. Cereb. Blood Flow. Metab. 33, 1493–1499. 10.1038/jcbfm.2013.128 23921897 PMC3790936

[B150] Sclip A. Tozzi A. Abaza A. Cardinetti D. Colombo I. Calabresi P. (2014). c-Jun N-terminal kinase has a key role in Alzheimer disease synaptic dysfunction *in vivo* . Cell Death Dis. 5, e1019. 10.1038/cddis.2013.559 24457963 PMC4040696

[B151] Sędzikowska A. Szablewski L. (2021). Insulin and insulin resistance in Alzheimer’s disease. Int. J. Mol. Sci. 22, 9987. 10.3390/ijms22189987 34576151 PMC8472298

[B152] Shah H. Albanese E. Duggan C. Rudan I. Langa K. M. Carrillo M. C. (2016). Research priorities to reduce the global burden of dementia by 2025. Lancet Neurol. 15, 1285–1294. 10.1016/S1474-4422(16)30235-6 27751558

[B153] Sharma M. K. Jalewa J. Hölscher C. (2014). Neuroprotective and anti-apoptotic effects of liraglutide on SH-SY5Y cells exposed to methylglyoxal stress. J. Neurochem. 128, 459–471. 10.1111/jnc.12469 24112036

[B154] Shehata M. K. Ismail A. A. Kamel M. A. (2023). Combined donepezil with astaxanthin via nanostructured lipid carriers effective delivery to brain for Alzheimer’s disease in rat model. Int. J. Nanomedicine 18, 4193–4227. 10.2147/IJN.S417928 37534058 PMC10391537

[B155] Shokouhi S. Taylor W. D. Albert K. Kang H. Newhouse P. A. Alzheimer’s Disease Neuroimaging Initiative (2020). *In vivo* network models identify sex differences in the spread of tau pathology across the brain. Alzheimers Dement. (Amst) 12, e12016. 10.1002/dad2.12016 32280740 PMC7144772

[B156] Simpson I. A. Chundu K. R. Davies-Hill T. Honer W. G. Davies P. (1994). Decreased concentrations of GLUT1 and GLUT3 glucose transporters in the brains of patients with Alzheimer’s disease. Ann. Neurol. 35, 546–551. 10.1002/ana.410350507 8179300

[B157] Stanley M. Macauley S. L. Holtzman D. M. (2016). Changes in insulin and insulin signaling in Alzheimer’s disease: cause or consequence? J. Exp. Med. 213, 1375–1385. 10.1084/jem.20160493 27432942 PMC4986537

[B158] Steen E. Terry B. M. Rivera E. J. Cannon J. L. Neely T. R. Tavares R. (2005). Impaired insulin and insulin-like growth factor expression and signaling mechanisms in Alzheimer’s disease-is this type 3 diabetes? J. Alzheimers Dis. 7, 63–80. 10.3233/jad-2005-7107 15750215

[B159] Stranahan A. M. Arumugam T. V. Cutler R. G. Lee K. Egan J. M. Mattson M. P. (2008). Diabetes impairs hippocampal function through glucocorticoid-mediated effects on new and mature neurons. Nat. Neurosci. 11, 309–317. 10.1038/nn2055 18278039 PMC2927988

[B160] Strauss S. Bauer J. Ganter U. Jonas U. Berger M. Volk B. (1992). Detection of interleukin-6 and alpha 2-macroglobulin immunoreactivity in cortex and hippocampus of Alzheimer’s disease patients. Lab. Invest. 66, 223–230. 1370967

[B161] Sun J. Chen S. Lu S. Zheng J. Deng Y. (2015). The effects of liraglutide on learning and memory in Alzheimer-like triple transgenic mice with type 2 diabetes. Tianjin Med. J. 43, 728–732.

[B162] Tadokoro K. Ohta Y. Inufusa H. Loon A. F. N. Abe K. (2020). Prevention of cognitive decline in Alzheimer’s disease by novel antioxidative supplements. Int. J. Mol. Sci. 21, 1974. 10.3390/ijms21061974 32183152 PMC7139972

[B163] Talbot K. (2014). Brain insulin resistance in Alzheimer’s disease and its potential treatment with GLP-1 analogs. Neurodegener. Dis. Manag. 4, 31–40. 10.2217/nmt.13.73 24640977 PMC4465775

[B164] Talbot K. Wang H.-Y. Kazi H. Han L.-Y. Bakshi K. P. Stucky A. (2012). Demonstrated brain insulin resistance in Alzheimer’s disease patients is associated with IGF-1 resistance, IRS-1 dysregulation, and cognitive decline. J. Clin. Invest. 122, 1316–1338. 10.1172/JCI59903 22476197 PMC3314463

[B165] Tillement L. Lecanu L. Papadopoulos V. (2011). Alzheimer’s disease: effects of β-amyloid on mitochondria. Mitochondrion 11, 13–21. 10.1016/j.mito.2010.08.009 20817045

[B166] Tiwari V. Kuhad A. Bishnoi M. Chopra K. (2009). Chronic treatment with tocotrienol, an isoform of vitamin E, prevents intracerebroventricular streptozotocin-induced cognitive impairment and oxidative-nitrosative stress in rats. Pharmacol. Biochem. Behav. 93, 183–189. 10.1016/j.pbb.2009.05.009 19464315

[B167] Trujillo J. M. Nuffer W. Smith B. A. (2021). GLP-1 receptor agonists: an updated review of head-to-head clinical studies. Ther. Adv. Endocrinol. Metab. 12, 2042018821997320. 10.1177/2042018821997320 33767808 PMC7953228

[B168] Ungar L. Altmann A. Greicius M. D. (2014). Apolipoprotein E, gender, and Alzheimer’s disease: an overlooked, but potent and promising interaction. Brain Imaging Behav. 8, 262–273. 10.1007/s11682-013-9272-x 24293121 PMC4282773

[B169] Urano F. Wang X. Bertolotti A. Zhang Y. Chung P. Harding H. P. (2000). Coupling of stress in the ER to activation of JNK protein kinases by transmembrane protein kinase IRE1. Science 287, 664–666. 10.1126/science.287.5453.664 10650002

[B170] Viña J. Lloret A. (2010). Why women have more Alzheimer’s disease than men: gender and mitochondrial toxicity of amyloid-beta peptide. J. Alzheimers Dis. 20 (2), S527–S533. 10.3233/JAD-2010-100501 20442496

[B171] Vlassara H. Uribarri J. (2014). Advanced glycation end products (AGE) and diabetes: cause, effect, or both? Curr. Diab Rep. 14, 453. 10.1007/s11892-013-0453-1 24292971 PMC3903318

[B172] Wang X. Wang L. Jiang R. Xu Y. Zhao X. Li Y. (2016a). Exendin-4 antagonizes Aβ1-42-induced attenuation of spatial learning and memory ability. Exp. Ther. Med. 12, 2885–2892. 10.3892/etm.2016.3742 27882091 PMC5103720

[B173] Wang X. Wang L. Xu Y. Yu Q. Li L. Guo Y. (2016b). Intranasal administration of Exendin-4 antagonizes Aβ31-35-induced disruption of circadian rhythm and impairment of learning and memory. Aging Clin. Exp. Res. 28, 1259–1266. 10.1007/s40520-016-0548-z 26920423

[B174] Wang Y. Chen S. Xu Z. Chen S. Yao W. Gao X. (2018). GLP-1 receptor agonists downregulate aberrant GnT-III expression in Alzheimer’s disease models through the Akt/GSK-3β/β-catenin signaling. Neuropharmacology 131, 190–199. 10.1016/j.neuropharm.2017.11.048 29223528

[B175] Watson G. S. Craft S. (2003). The role of insulin resistance in the pathogenesis of Alzheimer’s disease: implications for treatment. CNS Drugs 17, 27–45. 10.2165/00023210-200317010-00003 12467491

[B176] Woodruff G. Young J. E. Martinez F. J. Buen F. Gore A. Kinaga J. (2013). The presenilin-1 ΔE9 mutation results in reduced γ-secretase activity, but not total loss of PS1 function, in isogenic human stem cells. Cell Rep. 5, 974–985. 10.1016/j.celrep.2013.10.018 24239350 PMC3867011

[B177] Wu M. Zhang M. Yin X. Chen K. Hu Z. Zhou Q. (2021). The role of pathological tau in synaptic dysfunction in Alzheimer’s diseases. Transl. Neurodegener. 10, 45. 10.1186/s40035-021-00270-1 34753506 PMC8579533

[B178] Xiong H. Zheng C. Wang J. J. Song J. Z. Zhao G. Shen H. (2013). The neuroprotection of liraglutide on alzheimer-like learning and memory impairment by modulating the hyperphosphorylation of tau and neurofilament proteins and insulin signaling pathways in mice. J. Alzheimers Dis. 37, 623–635. 10.3233/jad-130584 24008687

[B179] Xiong X. Lu W. Qin X. Luo Q. Zhou W. (2020). Downregulation of the GLP-1/CREB/adiponectin pathway is partially responsible for diabetes-induced dysregulated vascular tone and VSMC dysfunction. Biomed. Pharmacother. 127, 110218. 10.1016/j.biopha.2020.110218 32559849

[B180] Yassine H. N. Solomon V. Thakral A. Sheikh-Bahaei N. Chui H. C. Braskie M. N. (2022). Brain energy failure in dementia syndromes: Opportunities and challenges for glucagon-like peptide-1 receptor agonists. Alzheimers Dement. 18, 478–497. 10.1002/alz.12474 34647685 PMC8940606

[B181] Yoon S. Y. Choi J. E. Yoon J. H. Huh J.-W. Kim D. H. (2006). BACE inhibitor reduces APP-beta-C-terminal fragment accumulation in axonal swellings of okadaic acid-induced neurodegeneration. Neurobiol. Dis. 22, 435–444. 10.1016/j.nbd.2005.12.013 16480887

[B182] Yu C.-J. Wang M. Li R.-Y. Wei T. Yang H.-C. Yin Y.-S. (2022). TREM2 and microglia contribute to the synaptic plasticity: from physiology to pathology. Mol. Neurobiol. 60, 512–523. 10.1007/s12035-022-03100-1 36318443

[B183] Yu C. J. Ma D. Y. Song L. L. Zhai Z. N. Tao Y. Zhang Y. (2020). The role of GLP-1/GIP receptor agonists in Alzheimer’s disease. Adv. Clin. Exp. Med. 29, 661–668. 10.17219/acem/121007 32614526

[B184] Zhang M. J. Wu Y. B. Gao R. N. Chen X. W. Chen R. Y. Chen Z. (2022). Glucagon-like peptide-1 analogs mitigate neuroinflammation in Alzheimer’s disease by suppressing NLRP2 activation in astrocytes. Mol. Cell. Endocrinol. 542, 111529. 10.1016/j.mce.2021.111529 34906628

[B185] Zhang Z. (2019). Protective effects of geniposide on behavioral and pathologic changes in APP/PS1 mice and exploration of molecular mechanism:down regulation of mTOR signal pathway and enhancement of autophagy. Dissertation. Taiyuan, Shanxi: Shanxi Medical University.

[B186] Zhao L. Wang L. Mo L. Sun Y. (2021). Effects of liraglutide on cognitive function and phosphorylation of Tau protein in streptozotocin-induced Alzheimer in rats. Carcinogenesis,Teratogenesis Mutagen. 33, 280–285.

[B187] Zheng J. (2022). Cerebral glucose metabolic phenotype mediates cognitive improving effect of glucagon like peptide-1 analogue in Alzheimer’s disease. Dissertation. Fuzhou, Fujian: Fujian Medical University.

[B188] Zheng M. Wang P. (2021). Role of insulin receptor substance-1 modulating PI3K/Akt insulin signaling pathway in Alzheimer’s disease. 3 Biotech. 11, 179. 10.1007/s13205-021-02738-3 PMC7981362 33927970

